# Hyperactivated glycolysis drives spatially patterned Kupffer cell depletion in MASLD

**DOI:** 10.7554/eLife.109206

**Published:** 2026-05-26

**Authors:** Jia He, Ran Li, Cheng Xie, Xiane Zhu, Keqin Wang, Zhao Shan

**Affiliations:** 1 Yunnan Key Laboratory of Cell Metabolism and Diseases, Center for Life Sciences, School of Life Sciences, Yunnan University Kunming China; Singapore Immunology Network Singapore; National Institute of Immunology India

**Keywords:** MASLD, Kupffer cells, glycolytic metabolism, metabolic reprogramming, cell death, Mouse

## Abstract

Metabolic dysfunction-associated steatotic liver disease (MASLD) progression is characterized by hepatic inflammation and cell death, yet the mechanisms underlying Kupffer cell (KC) loss remain poorly understood. Here, we sought to elucidate the metabolic basis of KC death during MASLD. Using metabolomics, immunostaining, and flow cytometry, we evaluated metabolic alterations and KC death throughout early MASLD progression. We found that KC death is an early hallmark of MASLD, exhibiting greater susceptibility and a spatial distribution consistent with KC zonation. Moreoever, KCs undergo progressive metabolic reprogramming toward enhanced glucose utilization during MASLD development, which is correlated with KC death. In combination with biochemical agonist, isotope tracing, and primary KC culture, we further demonstrated that augmented glycolytic metabolism directly drives KC death in vitro. Consistently, using *Chi3l1*-deficient mice, we further demonstrated that increased glucose utilization accelerates KC death in vivo. Together, these findings establish a causal link between glycolytic activation and KC loss during MASLD progression, highlighting glucose metabolic pathways as potential therapeutic targets to preserve KC homeostasis and mitigate MASLD.

## Introduction

Metabolic dysfunction-associated steatotic liver disease (MASLD) emerges as a prevalent liver condition in the Western world, affecting roughly one-third of the population (Hardy et al., 2016; Younossi et al., 2018; Younossi, 2019). Its incidence continues to climb due to its strong association with obesity, type 2 diabetes, and the metabolic syndrome (Eslam et al., 2020a; Rinella and Sanyal, 2016; Sheka et al., 2020). MASLD includes a spectrum of liver disorders, from metabolic dysfunction-associated fatty liver, often referred to as steatosis, to metabolic dysfunction-associated steatohepatitis (MASH) (Eslam et al., 2020b). MASH, characterized by steatosis, inflammation, ballooning injury, and varying degrees of fibrosis, marks the initial critical stage of MASLD (Friedman et al., 2018). Kupffer cells (KCs) are liver-resident macrophages located in the hepatic sinusoids (Gomez Perdiguero et al., 2015; Kazankov et al., 2019). Derived from the embryonic yolk sac, KC can self-renew through proliferation during adult homeostasis (Hashimoto et al., 2013). Studies suggest that KC contribute to triglyceride (TG) storage during MASH progression, as demonstrated by depleting CD207^+^ KC in CD207-DTR mice using diphtheria toxin or by using CD207^∆Bcl2l1^ mice to stimulate a low embryo-derived KC (EmKC) status (Tran et al., 2020). Upon KC death, monocyte-derived macrophages (MoMFs) gradually seed the KC pool and eventually replace the deceased KC (Scott et al., 2016). These MoMFs tend to be more inflammatory than EmKC, altering the liver’s response in MASH, eventually limiting TG storage and contributing to liver fibrosis (Daemen et al., 2021; Tran et al., 2020). Researchers, including our team, have observed a gradual decline in KCs during MASLD development (Daemen et al., 2021; He et al., 2025; Remmerie et al., 2020; Tacke, 2017; Tran et al., 2020). However, little is known regarding the dynamic loss of KCsand metabolic changes behind KC death during MASLD.

Emerging evidence highlights a fundamental role of glucose metabolism in regulating macrophage function, polarization, and survival (Du et al., 2019; Faas et al., 2021; Ma et al., 2020; Woods et al., 2022). Metabolic reprogramming is a well-established hallmark of macrophage activation and functional adaptation across diverse contexts (Murray et al., 2014). Within the unique metabolic milieu of the MASLD liver, characterized by lipotoxicity, insulin resistance, and disrupted nutrient fluxes, it is plausible that KC metabolism is profoundly altered. However, whether these metabolic perturbations directly contribute to the observed loss of KCs, and through which specific metabolic pathways, remains unclear. Therefore, this study aims to investigate the role of glucose metabolism in governing KC susceptibility to death during MASLD progression. We sought to define the glucose metabolic alterations that occur in KCs as MASLD develops and to establish a causal link between metabolic reprogramming and KC loss. Elucidating these mechanisms is essential for advancing our understanding of MASLD pathogenesis and for identifying potential therapeutic targets to preserve KC homeostasis and mitigate disease progression.

## Results

### The death of KCs is a pathological characteristic during MASLD

To systematically investigate KC death, we established an MASLD mouse model by feeding wild-type (WT) C57Bl/6J mice a high-fat high-cholesterol diet (HFHC) (Figure 1—figure supplement 1A). We then performed hematoxylin and eosin (H&E), Oil Red O, and Sirius Red staining to assess immune cell infiltration, fat accumulation, and liver fibrosis in mice fed HFHC for 0, 4, or 16 weeks. After 4 weeks of HFHC feeding, we observed a slight increase in lipid droplets, with no apparent signs of liver inflammation or fibrosis in hepatocytes (Figure 1—figure supplement 1A). By 16 weeks of HFHC feeding, both lipid droplets and immune cell infiltration had increased significantly, though liver fibrosis—as indicated by Sirius Red staining, which labels collagen deposition—was still not induced, and MASLD activity score remained below 4 (Figure 1—figure supplement 1A). Moreover, the body weight of mice fed HFHC gradually increased over the feeding period (Figure 1—figure supplement 1B). Analysis of serum alanine aminotransferase (ALT), aspartate aminotransferase (AST), serum and liver cholesterol, or liver TG levels revealed significant increases compared to mice fed a normal chow diet (NCD), except for serum TG, which was similar between NCD and HFHC-fed mice (Figure 1—figure supplement 1C). These findings collectively indicate the successful establishment of an early MASLD mouse model, without fibrosis development.

**Figure 1.**
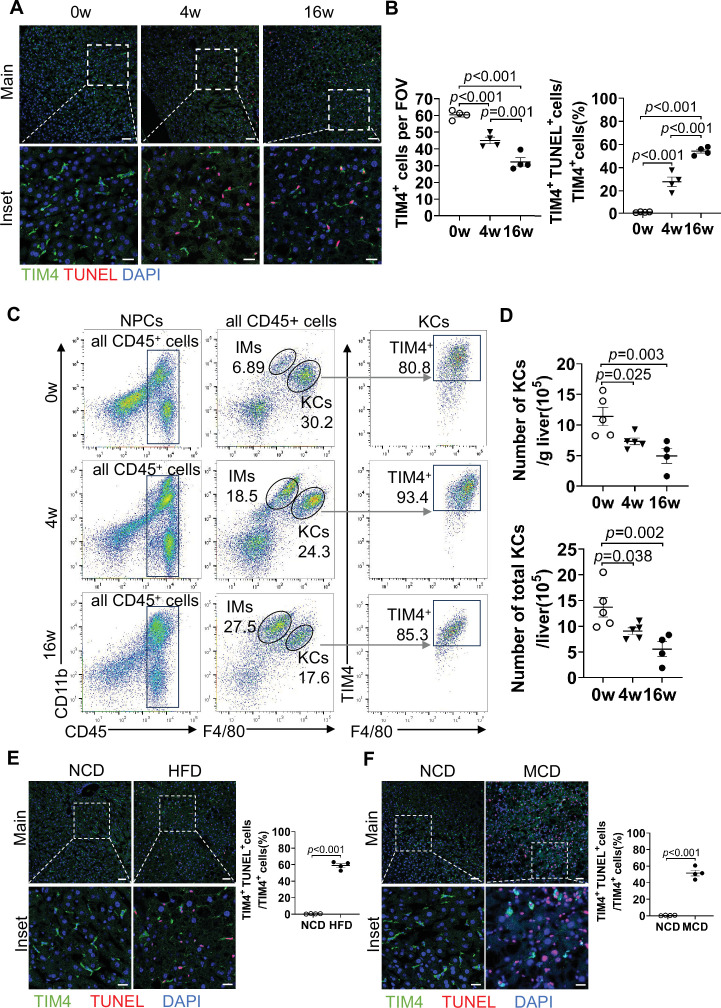
Kupffer cell (KC) death is a characteristic feature of metabolic dysfunction-associated steatotic liver disease (MASLD) progression. (**A–D**) Male wild-type C57BL/6J mice were fed a high-fat high-cholesterol diet (HFHC) for 0, 4, or 16 weeks. (**A**) KC death was assessed by immunostaining of liver sections for TIM4 (KC marker, red), TdT-mediated dUTP Nick-End Labeling (TUNEL) (green), and DAPI (nuclei, blue). Scale bar: 50 µm (main panels) and 20 µm (inset). (**B**) KC death was quantified. n=4 mice/group. (**C**) Flow cytometry analysis of KCs (CD45^+^ F4/80^hi^ CD11b^low^ TIM4^+^) and infiltrating macrophages (IMs) (CD45^+^ F4/80^low^CD11b^hi^ TIM4^-^) among isolated nonparenchymal cells (NPCs). (**D**) KC counts were quantified. n=4–5 mice/group. (**E–F**) Male wild-type C57BL/6J mice were fed either: (**E**) normal chow diet (NCD) or high-fat diet (HFD) for 20 weeks, or (**F**) NCD or methionine-choline-deficient diet (MCD) for 6 weeks. KC death was assessed by immunostaining of liver sections for TIM4 (green), TUNEL (red), and DAPI (nuclei, blue). Scale bar: 50 µm (main panels) and 20 µm (inset). KC death was quantified. n=4 mice/group. Representative images are shown in A, C, E, F. One-way ANOVA (**B, D**). Unpaired Student’s t-test (**E, F**). p-Value as indicated. Figure 1—source data 1.Numerical data of Figure 1B–D and E–F.

Subsequently, we investigated KC death by labeling KCs with antibodies targeting T cell immunoglobulin mucin protein 4 (TIM4) (Scott et al., 2018; Tran et al., 2020) and employing TdT-mediated dUTP Nick-End Labeling (TUNEL) to detect dead cells. Compared to baseline (0 week, prior to HFHC), KCs are significantly reduced with MASLD progression. Approximately 25% and nearly 60% of KCs underwent cell death by 4 and 16 weeks post-HFHC feeding, respectively (Figure 1A and B). To further validate KC death during MASLD progression, we performed co-staining of the apoptotic marker Cleaved caspase 3 (Cl-Casp3) with C-type lectin domain family 4 (Clec4f), another marker specific for KCs (Scott et al., 2016). Although the ratio of Cl-Casp3^+^/Clec4f^+^ was relatively lower, its occurrence and gradual increase followed a trend similar to that of the TUNEL staining (Figure 1—figure supplement 2A). Considering the potential recruitment of MoMFs into the liver, some of which may acquire KC features, including TIM4 and Clec4f expression (Daemen et al., 2021; Tran et al., 2020), we performed flow cytometry analysis of liver nonparenchymal cells (NPCs) to distinguish resident KCs from monocyte-derived populations. Resident KCs were identified as CD45^+^ F4/80^hi^ CD11b^low^ TIM4^hi^ cells, MoKCs as CD45^+^ F4/80^hi^ CD11b^low^ TIM4^low^ cells, and infiltrating macrophages (IMs) as CD45^+^ F4/80^+^ CD11b^hi^ TIM4^-^ cells (Figure 1C). Our analysis showed that all TIM4^+^ KCs were of EmKCs, with no detectable MoKCs at any time point examined (Figure 1C). Consistent with the immunostaining results, the number of resident KCs decreased as early as 4 weeks and continued to decline through 16 weeks of HFHC feeding (Figure 1C and D). In contrast, IMs progressively accumulated during HFHC feeding (Figure 1C and D). To further refine the identification of MoMFs, we exclude CD45^+^ F4/80^+^ CD11b^hi^ TIM4^-^ Ly6G^+^ neutrophils from the IM gate. This refined analysis yielded similar results, confirming a gradual increase in MoMFs over the course of HFHC feeding (Figure 1—figure supplement 2B and C).

**Figure 1—figure supplement 1.**
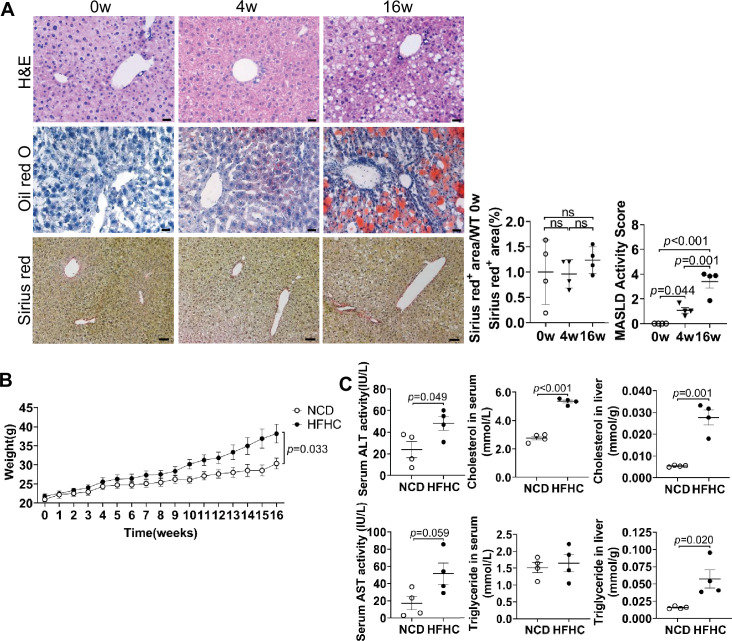
The generation of high-fat high-cholesterol diet (HFHC)-induced metabolic dysfunction-associated steatotic liver disease (MASLD) mouse model. Male wild-type C57B/6J mice were fed with HFHC for 0, 4, or 16 weeks. (**A**) Hematoxylin and eosin (H&E) (top), Oil Red O (middle), and Sirius Red staining (bottom) were performed to detect MASLD progression in liver sections at 0, 4, or 16 weeks after HFHC, respectively. Scale bar: 20 μm. Liver fibrosis was quantified. MASLD activity score is diagnosed. (**B**) Body weight was recorded during HFHC feeding. (**C**) Serum ALT, AST, cholesterol, triglyceride, or liver cholesterol triglyceride is measured at 16 weeks after HFHC. n=4 mice/group. Unpaired Student’s t-test (**B, C**); one-way ANOVA (**A**). p-Value as indicated. ns: not significant. Figure 1—figure supplement 1—source data 1.Numerical data of Figure 1—figure supplement 1A–C.

**Figure 1—figure supplement 2.**
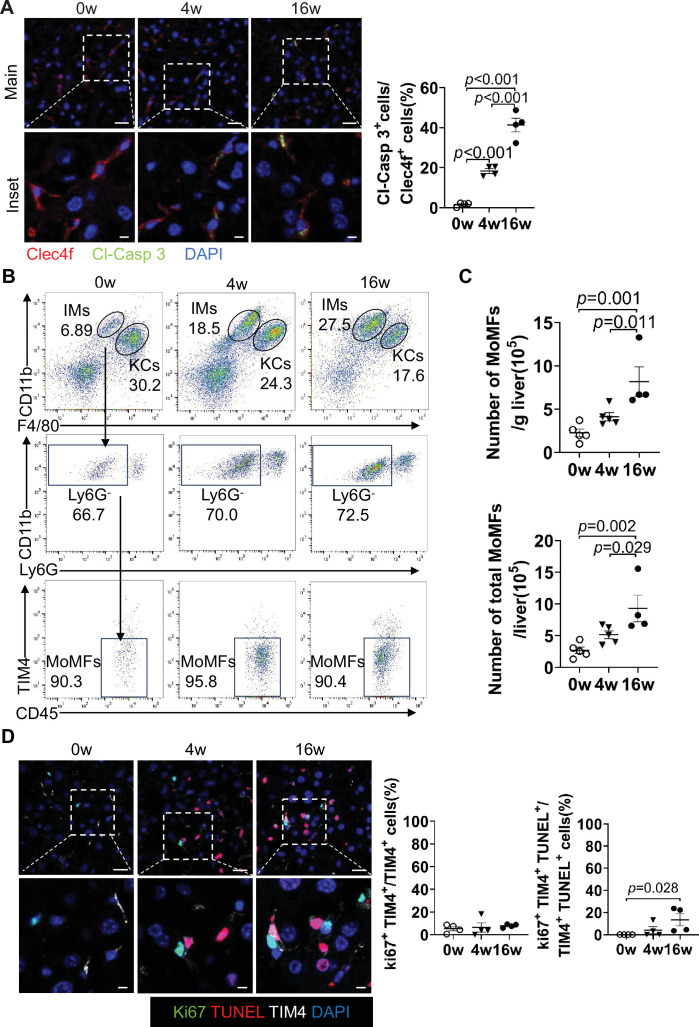
Examination of Kupffer cell (KC) death and monocyte-derived macrophages (MoMFs) recruitment in high-fat high-cholesterol diet (HFHC) mice. Male wild-type (WT) C57B/6J mice were fed with HFHC for 0, 4, or 16 weeks. (**A**) KC death was examined by immunofluorescence staining of Clec4f and cleavaged caspase 3 (Cl-Casp3) and DAPI (nuclei) in livers of mice. KC death was quantified. n=4 mice/group. (**B**) Flow cytometry analysis of MoMFs (CD45^+^Ly6G⁻CD11b^+^F4/80^low^TIM4^low/-^) among nonparenchymal cells (NPCs) of WT mice fed HFHC diet for 0, 4, or 16 weeks. (**C**) MoMFs counts were quantified. n=4–5 mice/group. (**D**) Proliferation-associated TdT-mediated dUTP Nick-End Labeling (TUNEL^+^) KCs was examined by co-staining of Ki67, TIM4, TUNEL, and DAPI (nuclei). Ki67^+^ KCs among TUNEL^+^ KCs was quantified. Representative images are shown in A, D. One-way ANOVA (**A, C, D**). p-Value as indicated. Scale bar: 20 μm (main panels), 5 μm (inset). Figure 1—figure supplement 2—source data 1.Numerical data of Figure 1—figure supplement 2A and C–D.

To exclude TUNEL false positivity associated with proliferation, we evaluated Ki67 expression in dying KCs (TUNEL^+^TIM4^+^). We observed that proliferating KCs (Ki67^+^TIM4^+^) were infrequent throughout HFHC feeding, and less than 15% of TUNEL^+^TIM4^+^ KCs co-expressed Ki67. This indicates that the majority of TUNEL^+^ KCs represent true cell death events rather than proliferation-related artifacts (Figure 1—figure supplement 2D). To examine the broader occurrence of this phenomenon in MASLD, we further assessed KC death in several other dietary mouse models, including mice fed a high-fat diet (HFD) for 20 weeks (Zhang et al., 2022) and a methionine/choline-deficient diet (MCD) for 6 weeks (Tran et al., 2020). Co-staining of TIM4 and TUNEL in liver sections revealed a notable increase in KC death in both the HFD and MCD groups compared to the control group fed an NCD (Figure 1E and F). These findings collectively confirm that progressive KC death is a pathological hallmark of MASLD observed across various dietary-induced models.

### KCs exhibit early and zone-specific susceptibility to death during MASLD progression

**Figure 2.**
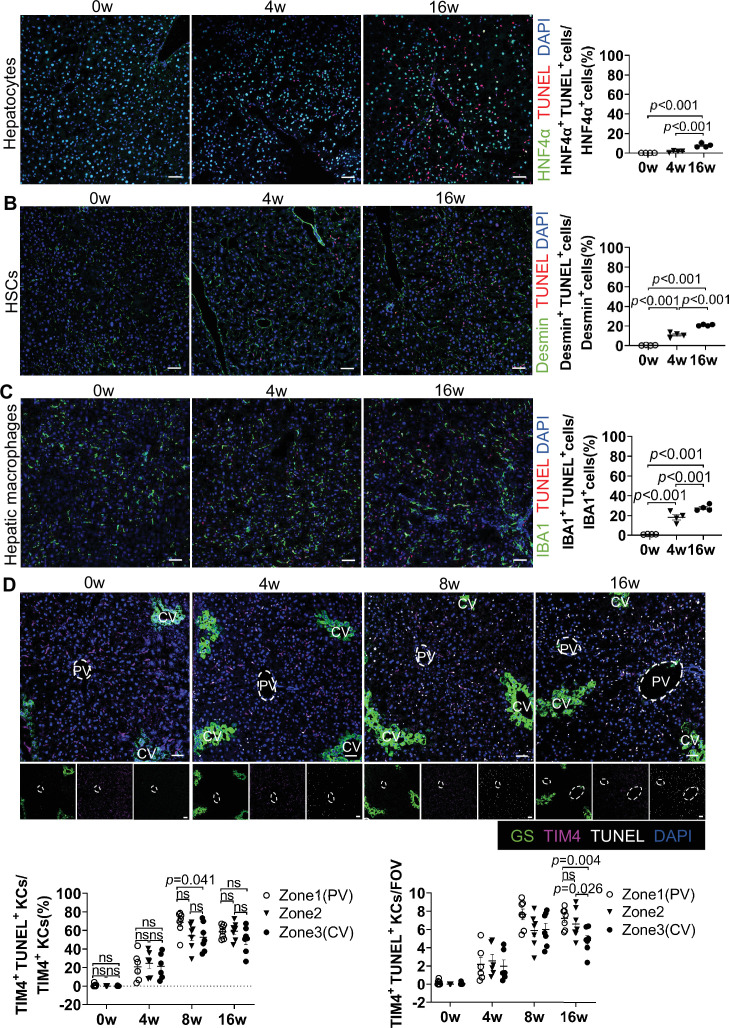
Kupffer cells (KCs) exhibit early and zone-specific susceptibility to death during metabolic dysfunction-associated steatotic liver disease (MASLD) progression. (**A–E**) Male wild-type C57BL/6J mice were fed a high-fat high-cholesterol diet (HFHC) for 0, 4, or 16 weeks. (**A–C**) Hepatic cell death was assessed by co-staining TdT-mediated dUTP Nick-End Labeling (TUNEL) with: (**A**) HNF4α (hepatocytes), (**B**) Desmin (hepatic stellate cells [HSCs]), (**C**) Iba1 (hepatic macrophages), and DAPI (nuclei, blue). Scale bars: 50 µm (main panels). Hepatic cell death was quantified (n=4 mice/group). (**D**) Zonal distribution of KC death was evaluated by co-staining TIM4 (KCs), TUNEL, glutamine synthetase (GS, central vein marker), and DAPI (nuclei, blue). Scale bars: 50 µm. Zonal distribution of KC death was quantified (n=6–7 mice/group). FOV: field of view. PV: portal vein. CV: central vein. Representative images are shown in A–D. One-way ANOVA (**A–D**). p-Value as indicated. Figure 2—source data 1.Numerical data of Figure 2A–D.

To compare relative susceptibility of different hepatic cell populations to MASLD-induced death, we performed TUNEL co-staining with lineage markers: HNF4α (hepatocytes), Desmin (hepatic stellate cells [HSCs]), and Iba1 (hepatic macrophages, including both KCs and MoMFs) (Figure 2A–C). Hepatocyte death showed minimal change during the initial 4 weeks but increased modestly by 16 weeks (Figure 2A). In contrast, HSC and hepatic macrophage mortality rose progressively throughout MASLD progression, with significant increases detectable as early as 4 weeks (Figure 2B and C). Notably, the finding that 60% of TIM4^+^ KCs are TUNEL^+^, compared to 30% of total Iba1^+^ cells, further supports that resident KCs undergo death more readily than the broader macrophage pool, which includes MoMFs.

We next determined whether KC death displays spatial zonation within the liver lobule. Based on the lobular architecture—comprising periportal (PP, zone 1), midzonal (Mid, zone 2), and pericentral (PC, zone 3) regions (Ben-Moshe et al., 2022)—we performed co-staining for TIM4, TUNEL, and glutamine synthetase (*GS; a central vein marker*) (Figure 2D). Spatial analysis revealed an early zonation bias in KC death. At 8 weeks of HFHC feeding, the proportion of TIM4^+^TUNEL^+^ cells among total TIM4^+^ KCs was significantly higher in PV regions compared to CV regions (p=0.041), indicating increased susceptibility of periportal KCs (Figure 2D). However, by 16 weeks, although absolute numbers of apoptotic KCs differed across zones, the proportional death rate became comparable (Figure 2D), suggesting that prolonged HFHC feeding leads to widespread KC loss that attenuates early zonation bias.

### KCs exhibit metabolic reprogramming with increased glycolysis during early MASLD

To define the glucose metabolic alterations that occur in KCs during MASLD development, we isolated KCs from WT mice at various time points of HFHC feeding (0, 4, 8, and 16 weeks). The purity of isolated KCs was confirmed by TIM4 immunofluorescence staining, with TIM4^+^ cells exceeding 90% (Figure 3—figure supplement 1A). We then conducted qRT-PCR to assess the mRNA expression levels of key enzymes involved in glycolysis (*Slc2a1, Hk3, Pfkfb3, Pkm*), the pentose phosphate pathway (PPP) (*G6pd, 6pdg*), glycogenolysis (*Pygl*), and glycogenesis (*Gys1, Ugp2*). Our data revealed that mRNA expression of rate-limiting enzymes for fast glucose metabolism, such as glycolysis and PPP, was significantly increased as early as 8 weeks after initiating the HFHC (Figure 3—figure supplement 1B). While glycolytic enzyme expression remained elevated, PPP enzyme expression began to decline by 16 weeks (Figure 3—figure supplement 1B). In contrast, enzymes linked to slower glucose metabolism, such as oxidative phosphorylation (*Idh1, Ogdh*), did not exhibit significant changes (Figure 3—figure supplement 1B). Furthermore, mRNA expression of glycogenesis and glycogenolysis rate-limiting enzymes started to decrease at 8 and 16 weeks, respectively, suggesting that glucose uptake becomes the primary source of KC glucose metabolism during this period (Figure 3—figure supplement 1B). Additionally, we investigated mRNA expression of rate-limiting enzymes involved in β-oxidation (*Acadm, Hadh*) but found no significant differences (Figure 3—figure supplement 1B). These data suggest a time-dependent metabolic inflexibility, where impaired glycogen handling and mitochondrial inertia drive a sustained glycolytic dependence in KCs, which may exacerbate their vulnerability (particularly in periportal zones, where glycogen storage is predominant; Okada et al., 2025).

**Figure 3.**
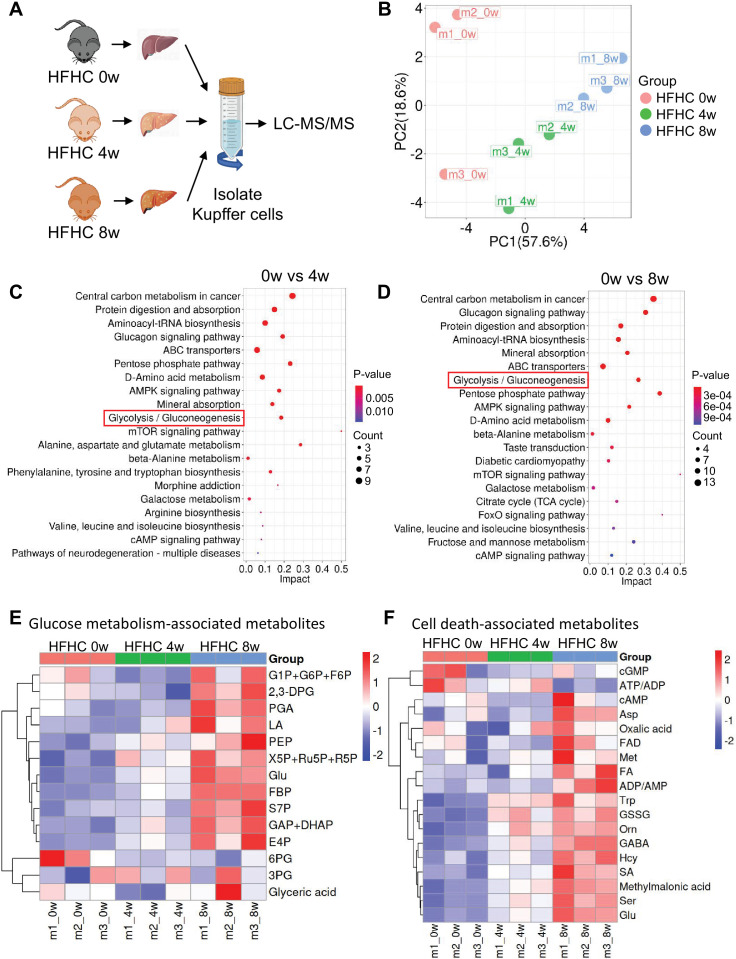
Kupffer cells (KCs) exhibit metabolic reprogramming with increased glycolysis during early metabolic dysfunction-associated steatotic liver disease (MASLD). (**A**) Experimental design for metabolomic analysis of KCs isolated from male wild-type mice fed a high-fat high-cholesterol diet (HFHC) for 0, 4, or 8 weeks. n=3 mice/group. (**B**) Principal component analysis (PCA) of enriched metabolites in KCs across different dietary durations. (**C–D**) Kyoto Encyclopedia of Genes and Genomes (KEGG) pathway enrichment analysis of metabolic pathways upregulated in KCs at 4 weeks (**C**) or 8 weeks (**D**). The glucose metabolism pathway is highlighted by red rectangles. (**E**) Heatmap depicting significantly altered metabolites involved in glucose metabolism pathways in KCs across different dietary durations. (**F**) Heatmap depicting significantly altered metabolites involved in cell death in KCs across different dietary durations. Figure 3—source data 1.Numerical data of Figure 3B–F.

To validate KC-specific metabolic alterations in MASLD, we performed metabolomic analysis on primary KCs from WT mice fed an HFHC for 0, 4, or 8 weeks (Figure 3A). Given that KC death peaked at 8 weeks in our model (Figure 2D), we focused on these early time points to capture initial metabolic shifts. Principal component analysis (PCA) of KC metabolites revealed distinct, diet duration-dependent clustering (Figure 3B). Kyoto Encyclopedia of Genes and Genomes (KEGG) pathway enrichment analysis identified glucose metabolism pathways—including glycolysis and the PPP—as the most significantly upregulated during MASLD progression (Figure 3C and D). This glycolytic activation was further corroborated by time-dependent accumulation of key intermediates, including glucose, phosphatidylethylamine (PEA), phosphoenolpyruvate (PEP), fructose-1,6-bisphosphate (FBP), and lactate (LA) in heatmap analysis (Figure 3E). Critically, we observed progressive increases in pro-apoptotic metabolites generated through these glucose metabolism pathways: redox disruptors (GSSG, FAD), mitochondrial toxins (methylmalonic acid), and apoptosis mediators (Hcy)—all exhibiting temporal coupling with glycolytic intermediates (Figure 3F). This demonstrates that KCs undergo rapid glycolytic reprogramming during early MASLD pathogenesis, which actively generates cytotoxic effectors coinciding with their peak vulnerability.

**Figure 3—figure supplement 1.**
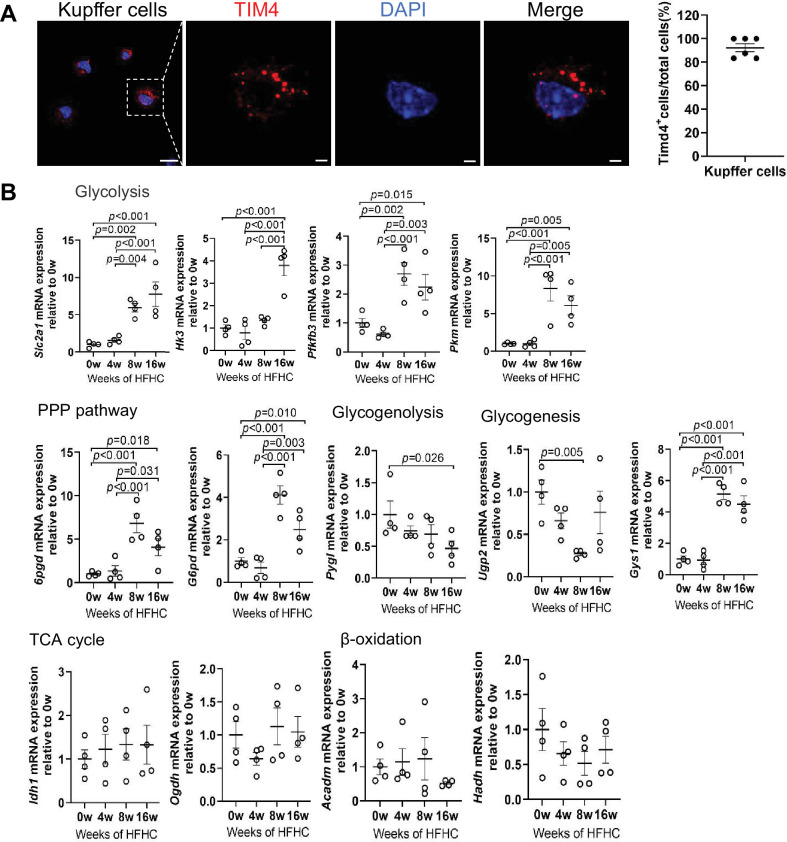
Dynamic changes in mRNA expression levels of rate-limiting enzyme genes involved in glucose metabolism. (**A**) Purity of isolated Kupffer cells (KCs) was examined by immunofluorescence staining of TIM4 and DAPI (nuclei). Scale bar: 20 μm (main panels), 5 μm (inset). Purity was quantified (n=6 independent experiments). (**B**) qRT-PCR analysis of mRNA expression levels of key rate-limiting enzymes in glycolysis, pentose phosphate pathway (PPP), glycogenolysis, glycogenesis, TCA cycle, and β-oxidation in wild-type (WT) KCs from mice for indicated dietary durations (n=4 mice/group). One-way ANOVA (**B**), p-values as indicated. Figure 3—figure supplement 1—source data 1.Numerical data of Figure 3—figure supplement 1A–B.

### Excessive glucose metabolic activity contributes to KC death

**Figure 4.**
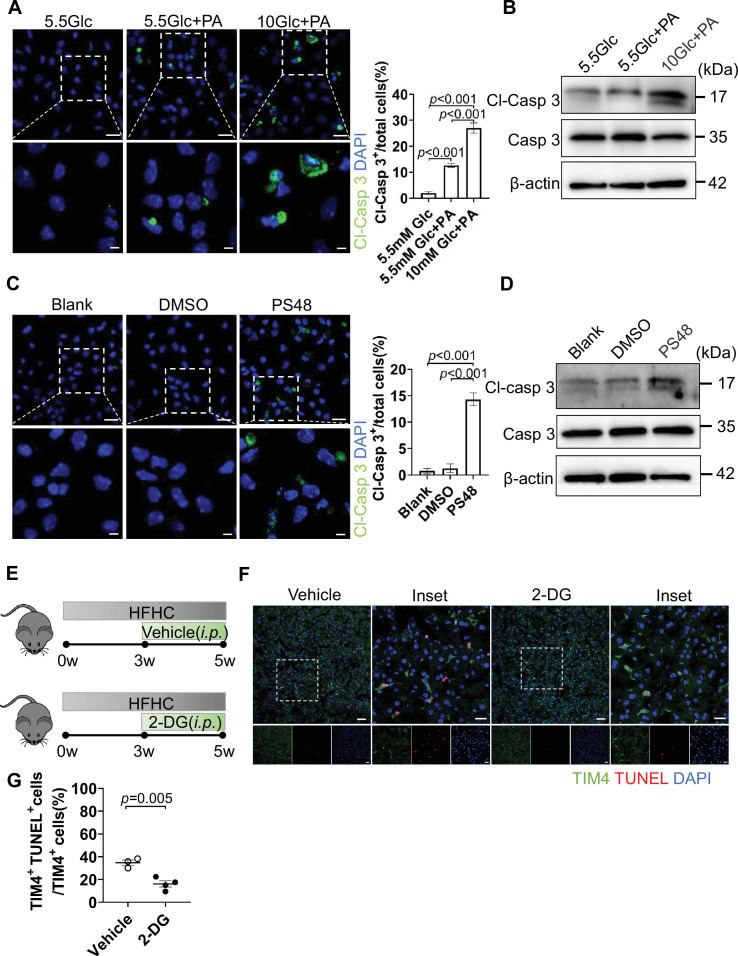
Excessive glucose metabolic activity contributes to Kupffer cell (KC) death. (**A–B**) Isolated KCs were treated for 24 hr with: 5.5 mM glucose+isopropanol (control), 5.5 mM glucose+800 µM palmitic acid (PA), 10 mM glucose+800 µM PA. Cell viability was assessed by Cleaved caspase 3 (Cl-Casp3) staining (Cl-Casp3^+^ cells = dead). Scale bars: 20 µm (main panels), 5 µm (insets). Cl-Casp3 was detected by western blot. (**C–D**) Isolated KCs were treated for 24 hr with: blank (no treatment), DMSO (vehicle control), 20 µM PS48 (PDK1 activator). Scale bars: 20 µm (main panels), 5 µm (insets). Cell death was analyzed as above. (**E**) Experimental design. Male wild-type (WT) mice were fed a high-fat high-cholesterol diet (HFHC) for 5 weeks. From the third week onward, mice received intraperitoneal injections of either vehicle or 2-DG (50 mg/kg) every other day (n=3–4 mice/group). (**F–G**) Effects of glycolysis inhibition on KC death after 5 weeks of HFHC feeding. (**F**) Representative images of liver sections co-stained with TdT-mediated dUTP Nick-End Labeling (TUNEL) and the KC marker TIM4. (**G**) Quantification of TUNEL^+^ KCs. Data are presented as mean ± SEM. Statistical analysis was performed using one-way ANOVA (**A, C**) and an unpaired Student’s t-test (**G**); p-values are indicated. Figure 4—source data 1.Numerical data of Figure 4A, C and G. Figure 4—source data 2.PDF file containing original western blots for Figure 4B and D, indicating the relevant bands and treatments. Figure 4—source data 3.Original files for western blot analysis displayed in Figure 4B and D.

To investigate the direct role of glucose metabolism in KC death, isolated KCs were subjected to in vitro metabolic perturbations. First, KCs were treated with a combination of high glucose and palmitic acid (PA) to model MASLD pathology. Physiological glucose (5.5 mM) combined with PA (800 µM) increased KC death by approximately 10%. Strikingly, glucose at a concentration mimicking HFHC feeding (10 mM) combined with PA (800 µM) increased KC death by approximately 27%. This was evidenced by increased Cl-Casp3 staining (Figure 4A) and elevated Cl-Casp3 protein levels via western blot (Figure 4B).

**Figure 4—figure supplement 1.**
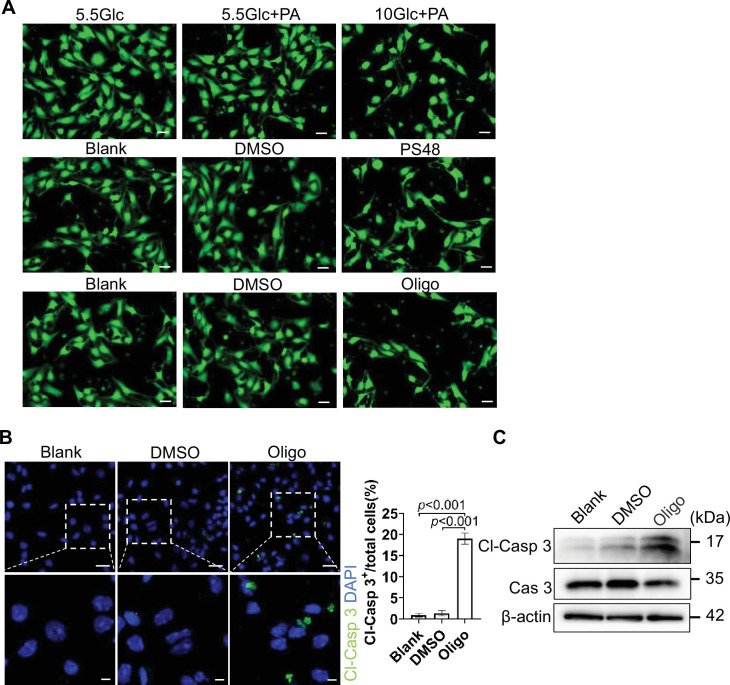
Excessive glucose metabolic activity contributes to Kupffer cell (KC) death. (**A**) Calcein-AM was used to assess primary KC viability treated as in main Figure 4. Scale bar: 20 μm. Representative images are shown. (**B–C**) Isolated KCs were treated for 24 hr with: blank (no treatment), DMSO (vehicle control), 20 µM oligomycin (Oligo, ATP synthase inhibitor). Scale bars: 20 µm (main panels), 5 µm (insets). Cell death was analyzed as above. One-way ANOVA (**B**). p-Value as indicated. Figure 4—figure supplement 1—source data 1.Numerical data of Figure 4—figure supplement 1B. Figure 4—figure supplement 1—source data 2.PDF file containing original western blots for Figure 4—figure supplement 1C, indicating the relevant bands and treatments. Figure 4—figure supplement 1—source data 3.Original files for western blot analysis displayed in Figure 4—figure supplement 1C.

Second, we treated KCs with the pyruvate dehydrogenase kinase (PDK1) activator PS48, which directly stimulates glycolysis. The results indicated a markedly increased KC death compared to blank or DMSO vehicle controls (Figure 4C and D). Finally, oligomycin (an ATP synthase inhibitor) was used to force glycolytic reliance by blocking mitochondrial ATP production. Oligomycin treatment similarly induced significant KC death (Figure 4—figure supplement 1A and B), phenocopying the effects of direct glycolytic activation. KCs are difficult to culture and prone to death in vitro. To assess the health status of KCs in our metabolic perturbation assays, we also examined KC viability using Calcein-AM staining, a fluorescent dye that labels metabolically active cells. Image analysis confirmed that the KCs were healthy and alive under all tested conditions (Figure 4—figure supplement 1C), indicating that our isolated KCs were suitable for in vitro experiments.

To determine whether enhanced glycolytic activity contributes to KC death in vivo during MASLD, we pharmacologically inhibited glycolysis using 2-deoxy-D-glucose (2-DG). 2-DG functions as a glucose analog that enters cells but cannot be fully metabolized, thereby inhibiting glycolysis through blockade of hexokinase and phosphoglucose isomerase (Figure 4E). WT C57BL/6J mice were fed an HFHC for 5 weeks to induce early-stage MASLD. To inhibit KC glycolysis in vivo, mice received intraperitoneal injections of either vehicle or 2-DG (50 mg/kg) every other day beginning at week 3 of HFHC feeding. The relatively short duration of HFHC exposure (5 weeks) combined with low-dose 2-DG treatment was designed to minimize potential confounding effects of systemic glycolytic inhibition on hepatocytes. At the end of the 5-week feeding period, KC survival was assessed by co-immunostaining liver sections for the KC marker TIM4 and TUNEL to detect apoptotic cells (Figure 4F). Quantitative analysis demonstrated that 2-DG-treated mice exhibited a significantly lower proportion of TUNEL^+^ KCs compared with vehicle-treated controls (Figure 4G). These results indicate that pharmacological inhibition of glycolysis protects KCs from cell death during MASLD. Together with our in vitro data showing that glycolytic activation promotes KC death.

### Enhanced glycolytic flux in *Chi3l1^-/-^* macrophages

**Figure 5.**
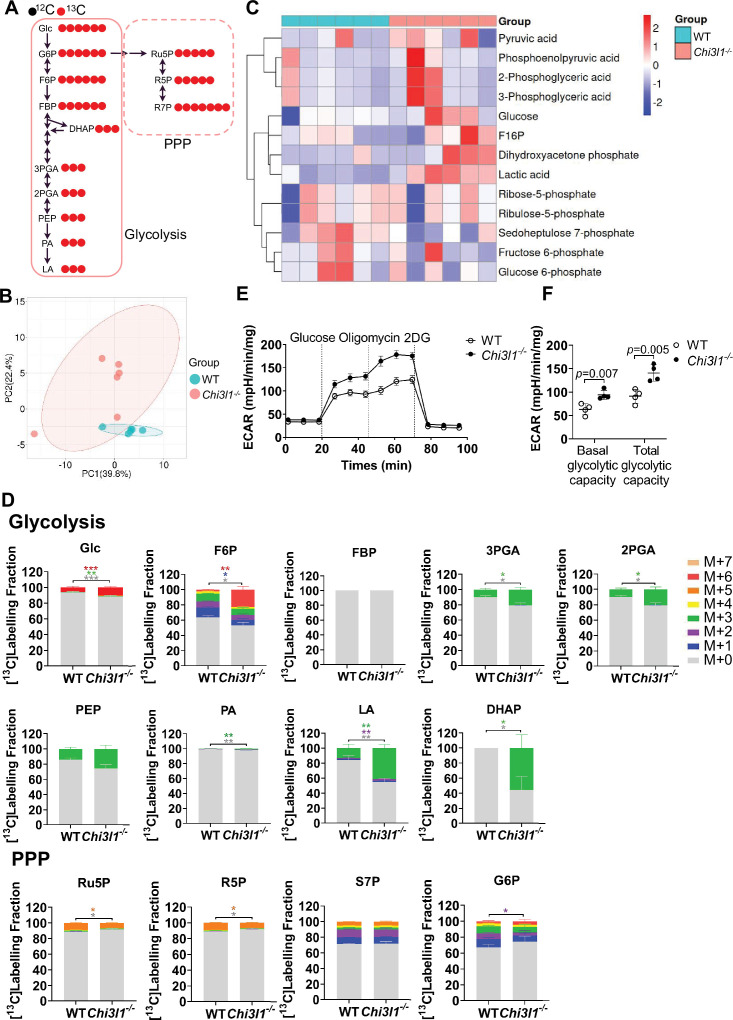
Enhanced glycolytic flux in *Chi3l1^-/-^* macrophages. (**A**) Schematic diagram depicting the fate of glucose-derived ribose carbons in wild-type (WT) mouse macrophages. (**B**) Principal component analysis (PCA) of metabolites in WT and *Chi3l1^-/-^* bone marrow-derived macrophages (BMDMs) cultured with [U-^13^C]glucose. (**C**) Heatmap depicting significantly altered glycolysis and pentose phosphate (PPP) metabolites in WT and *Chi3l1^-/-^* BMDMs. (**D**) Glucose metabolic flux analysis in WT and *Chi3l1^-/-^* BMDMs cultured with [U-^13^C]glucose showing mass isotopologue distributions of: glycolytic intermediates (Glc, F6P, FBP, 3PGA, 2PGA, PEP, PA, LA, G6P) and PPP intermediates (Ru5P, R5P, S7P, DHAP). Data represent n=6 biological replicates/group. (**E–F**) Extracellular acidification rate (ECAR) analysis of WT or *Chi3l1^-/-^* BMDM cells. BMDMs were sequentially treated with glucose, oligomycin, and 2-DG as indicated during seahorse. Unpaired Student’s t-test (**D, F**). *p<0.05, **p<0.01, ***p<0.001. Figure 5—source data 1.Numerical data of Figure 5B–F.

To investigate whether hyperactivated glycolysis specifically in KCs drives their death *in vivo*, we focused on Chitinase 3-like 1 (Chi3l1; gene *Chi3l1*)—a known inhibitor of glucose uptake (He et al., 2025). Since Chi3l1 suppresses glucose uptake by KCs, KCs in *Chi3l1^-/-^* mice are expected to maintain a state of hyperactivated glycolysis. To confirm this, we first performed a glucose metabolic flux assay in KCs. However, due to the limited availability of primary KCs, we used bone marrow-derived macrophages (BMDMs) to dissect the Chi3l1-dependent metabolic mechanisms. Uniformly labeled [U-^13^C]glucose tracer analysis (Figure 5A) revealed genotype-specific metabolic reprogramming: PCA showed distinct clustering between *Chi3l1^-/-^* and WT BMDMs (Figure 5B), and heatmaps demonstrated pronounced accumulation of glycolytic intermediates (Figure 5C). Metabolic flux quantification confirmed significantly elevated U-^13^C enrichment in glycolytic metabolites—including glucose (Glc), fructose-6-phosphate (F6P), 3-phosphoglycerate (3PGA), 2-phosphoglycerate (2PGA), PEP, pyruvate (PA), and LA—whereas glucose-6-phosphate (G6P), FBP, and PPP intermediates (ribulose-5-phosphate [Ru5P], ribose-5-phosphate [R5P], sedoheptulose-7-phosphate [S7P]) remained unchanged. Dihydroxyacetone phosphate (DHAP) was the only PPP-linked metabolite showing increased enrichment (Figure 5D), indicating that *Chi3l1* deletion selectively hyperactivates glycolysis without engaging the PPP. Furthermore, extracellular acidification rate (ECAR) analysis also showed significantly elevated glycolytic capacity in *Chi3l1^-/-^* BMDMs (Figure 5E and F).

**Figure 5—figure supplement 1.**
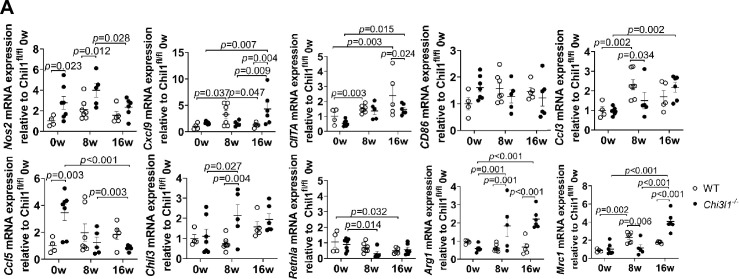
Comparison of Kupffer cell (KC) polarization between wild-type (WT) and *Chi3l1^-/-^* mice during metabolic dysfunction-associated steatotic liver disease (MASLD) progression. (**A**) qPCR analysis of key genes involved in macrophage polarization pathway in liver tissues of WT and *Chi3l1^-/-^* mice fed with high-fat high-cholesterol diet (HFHC) at indicated week. n=4–7 mice/group. Representative images are shown in A. One-way ANOVA (**A**). p-Value as indicated. Figure 5—figure supplement 1—source data 1.Numerical data of Figure 5—figure supplement 1A.

**Figure 5—figure supplement 2.**
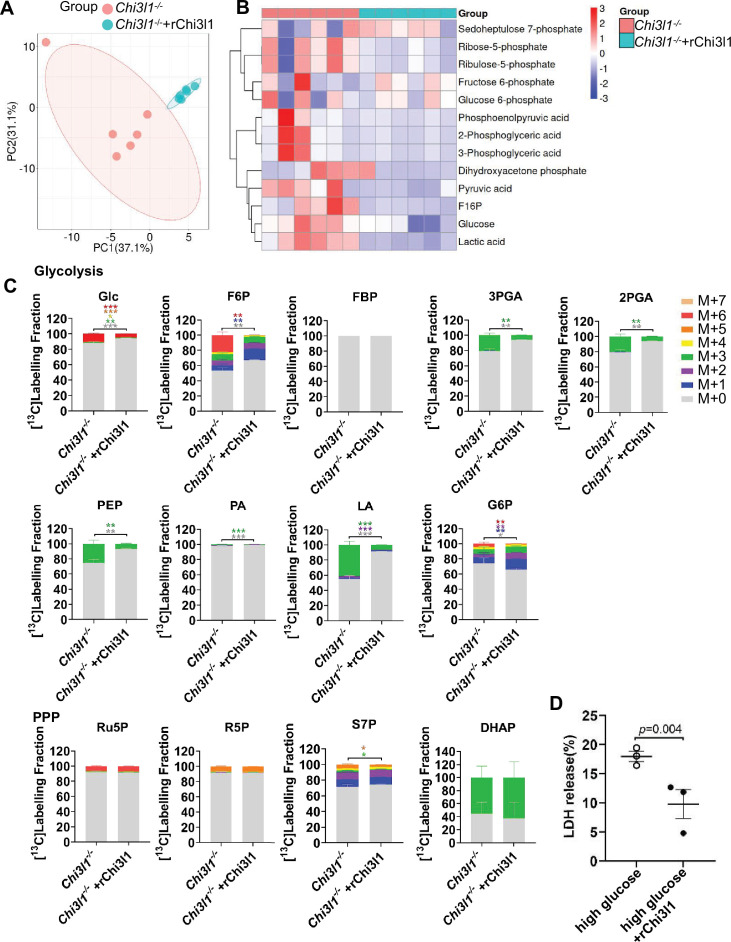
Recombinant Chi3l1 (rChi3l1) inhibits glucose utilization in *Chi3l1^-/-^* bone marrow-derived macrophages (BMDMs). (**A**) Comparative principal component analysis (PCA) of metabolites in *Chi3l1^-/-^* and *Chi3l1^-/-^* supplemented with rChi3l1 BMDMs cultured with uniformly labeled [U-^13^C]glucose. (**B**) Heatmap depicting significantly altered glycolysis and pentose phosphate pathway (PPP) metabolites in *Chi3l1^-/-^* and *Chi3l1^-/-^* supplemented with rChi3l1 BMDMs. (**C**) Glucose metabolic flux analysis in *Chi3l1^-/-^* and *Chi3l1^-/-^* supplemented with rChi3l1 BMDMs showing mass isotopologue distributions of: glycolysis pathway intermediates (Glc, F6P, FBP, 3PGA, 2PGA, PEP, PA, LA, G6P) and PPP intermediates (Ru5P, R5P, S7P, DHAP). Data represent n=6 biological replicates. (**D**) Lactate dehydrogenase (LDH) activity in culture medium of *Chi3l1^-/-^* BMDMs treated for 24 hr with: 10 mM glucose (high glucose), 10 mM glucose+100 ng/mL rChi3l1. Unpaired Student’s t-test (**D**). p-Value as indicated. Figure 5—figure supplement 2—source data 1.Numerical data of Figure 5—figure supplement 2A–D.

Increased glycolysis is often associated with proinflammatory polarization of macrophages (Tacke, 2017). To assess the effect of *Chi3l1* loss on KC polarization, we further isolated KCs from WT and *Chi3l1^-/-^* mice fed an HFHC at 0, 8, and 16 weeks, and measured the mRNA expression of proinflammatory markers (*Nos2, Cxcl9, Ciita, Cd86, Ccl3,* and *Ccl5*) and anti-inflammatory markers (*Chil3, Retnla, Arg1,* and *Mrc1*). In WT KCs, the mRNA levels of proinflammatory markers increased with MASLD progression: *Nos2, Cxcl9, CIITA,* and *Ccl3* rose significantly, while *CD86* and *Ccl5* showed an upward trend (Figure 5—figure supplement 1A). In contrast, *Chi3l1^-/-^* KCs exhibited higher baseline expression of proinflammatory genes, with *Nos2* and *Ccl5* significantly elevated and *Cxcl9* and *CD86* showing an increasing tendency compared to WT. As MASLD developed, proinflammatory gene expression increased further in *Chi3l1^-/-^* KCs. No significant differences in anti-inflammatory marker expression were observed between WT and *Chi3l1^-/-^* KCs at baseline. However, with MASLD progression, *Chi3l1^-/-^* KCs displayed a rising trend in the expression of anti-inflammatory markers, including *Chil3, Arg1,* and *Mrc1* (Figure 5—figure supplement 1A). Together, these data suggest that Chi3l1 deficiency drives KCs toward a partially proinflammatory phenotype.

Since Chi3l1 primarily functions in its secretory form as a macrophage glucose uptake inhibitor (He et al., 2025), we also added recombinant Chi3l1 (rChi3l1) supplementation group. rChi3l1 supplementation reversed the hyper-glycolytic flux, restoring it to levels comparable to those in WT macrophages (Figure 5—figure supplement 2A–D). This was accompanied by reduced mass isotopologue distributions of key glycolytic intermediates (Glc, F6P, FBP, 3PGA, 2PGA, PEP, PA, LA, G6P) (Figure 5—figure supplement 2B and C). In contrast, PPP intermediates (Ru5P, R5P, S7P, DHAP) were unaffected (Figure 5—figure supplement 2B and C), confirming the specificity of the effect on glycolysis. Functionally, rChi3l1 significantly lowered lactate dehydrogenase (LDH) activity in high glucose-treated BMDMs, further corroborating the attenuation of glycolytic output (Figure 5—figure supplement 2D). We acknowledge that detailed metabolic flux analysis was performed in BMDMs rather than primary KCs due to the technical challenge of obtaining sufficient viable cells for such assays. While ontogenically distinct from KCs, BMDMs are a widely accepted model for mechanistic metabolic dissection. Importantly, the hyperglycolytic phenotype observed in *Chi3l1^-/-^* BMDMs was consistent with our in vivo KC data: Chi3l1 deficiency drove proinflammatory polarization. Thus, despite this limitation, the concordance between our in vitro mechanistic findings and in vivo phenotypic data provides compelling evidence that *Chi3l1^-/-^* mice serve as a mechanistic model to investigate glycolysis-driven KC death during MASLD. Collectively, these data establish that Chi3l1 reduces glycolytic flux—without affecting PPP activity—in macrophages, thereby providing a mechanistic model to investigate glycolysis-driven KC death.

### Enhanced glycolysis drives KC death in MASLD

**Figure 6.**
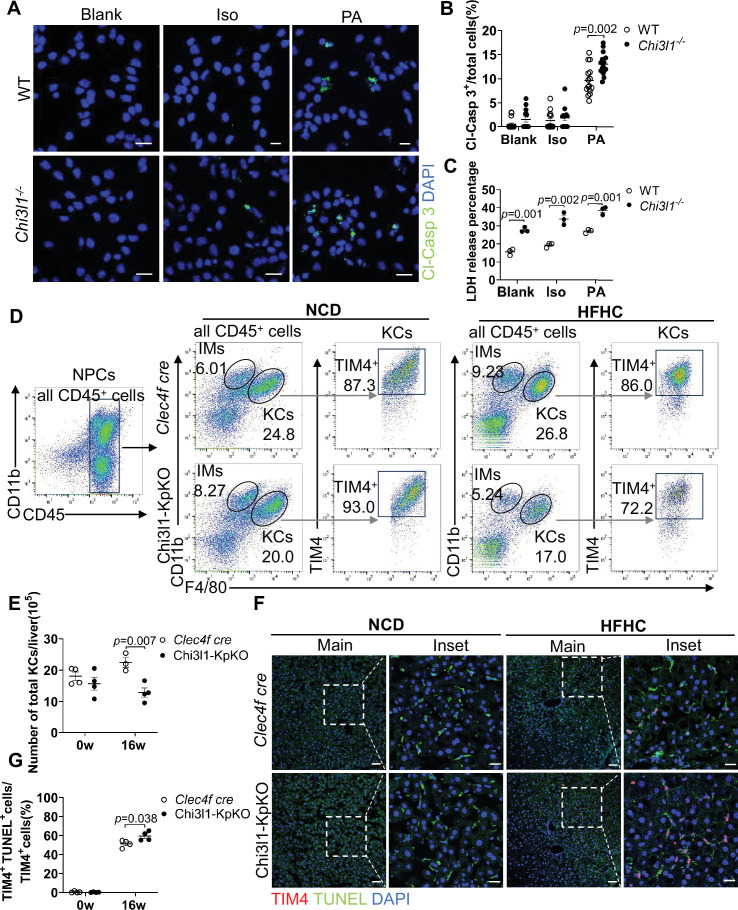
Enhanced glycolysis accelerated Kupffer cell (KC) death during metabolic dysfunction-associated steatotic liver disease (MASLD). (**A**) Cleaved caspase 3 (Cl-Casp3) staining to detect wild-type (WT) and *Chi3l1^-/-^* KC death. Cells were under treatment without (blank) or with either isopropyl alcohol (Iso) or palmitic acid (PA) for 24 hr. Scale bar: 20 μm. (**B**) Cl-Casp3^+^ cells were quantified. (**C**) Lactate dehydrogenase (LDH) release measurement in culture medium of KCs isolated from male WT and *Chi3l1^-/-^* mice was measured after treatment for 24 hr with: blank (no treatment), ISO (vehicle control), 800 µM PA. (**D**) Flow cytometry analysis of KCs (CD45^+^ F4/80^hi^ CD11b^low^ TIM4^+^) and monocyte-derived macrophages (MoMFs) (CD45^+^ F4/80^low^ CD11b^hi^ TIM4^-^) among nonparenchymal cells (NPCs) in *Clec4f-cre* and Chi3l1-KpKO mice fed high-fat high-cholesterol diet (HFHC) for 0 or 16 weeks. (**E**) KC counts were quantified. n=4 mice/group. (**F**) KC death was assessed by immunostaining of TIM4 (KC marker, green), TdT-mediated dUTP Nick-End Labeling (TUNEL) (red), and DAPI (nuclei, blue) in liver sections from *Clec4f-cre* and Chi3l1-KpKO mice fed HFHC for 0 or 16 weeks. Scale bar: 50 μm (main panels) and 20 μm (inset). (**G**) KC death was quantified. n=4 mice/group. Representative images shown (**A, D, F**). Unpaired Student’s t-test (**B, C, E, G**). p-Value as indicated. Figure 6—source data 1.Numerical data of Figure 6B–C, E and G.

We therefore employed *Chi3l1^-/-^* mice as a mechanistic model to investigate glycolysis-driven KC death. First, we isolated primary KCs from WT and *Chi3l1^-/-^* mice and treated them with PA or Iso, or left untreated (blank). *Chi3l1^-/-^* KCs exhibited significantly increased susceptibility to PA-induced cell death compared to WT controls in vitro, as indicated by elevated Cl-Casp3 immunostaining (Figure 6A and B). This result was further confirmed by increased LDH release from *Chi3l1^-/-^* KCs (Figure 6C). To delineate the cellular source of Chi3l1 in MASLD livers, we performed immunostaining on serially sectioned liver tissues from mice fed an HFHC for 16 weeks. Consecutive sections were independently probed for Chi3l1 or lineage-specific markers (HNF4α, Desmin, Iba1), enabling cellular localization through morphological alignment across sequential slices. This revealed predominant Chi3l1 expression in hepatic macrophages (Figure 6—figure supplement 1A). Moreover, Chi3l1 expression was significantly elevated in KCs (F4/80^+^TIM4^+^) of HFHC-fed mice, compared to NCD controls (Figure 6—figure supplement 1B), suggesting Chi3l1 is primarily expressed in KCs and may function as a KC-autonomous regulator.

**Figure 6—figure supplement 1.**
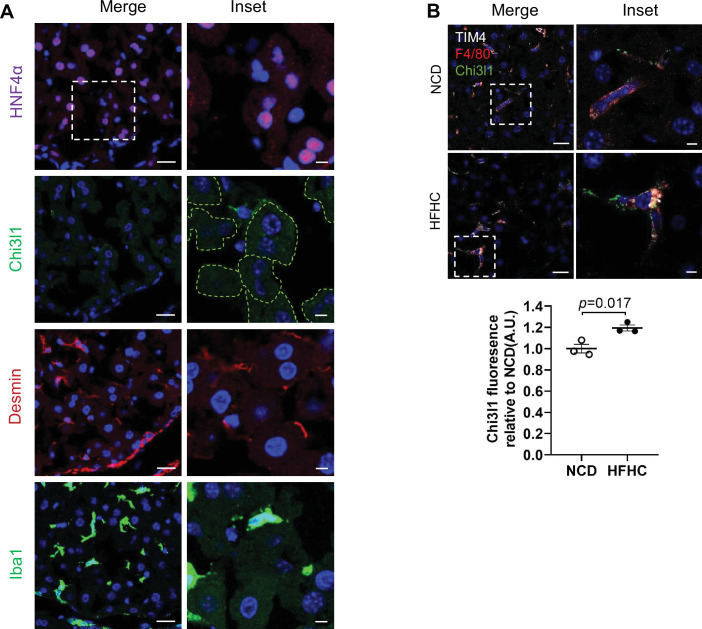
Chi3l1 is majorly expressed in hepatic macrophages, and its expression is upregulated during metabolic dysfunction-associated steatotic liver disease (MASLD). (**A**) Cellular source of Chi3l1 was assessed by immunohistochemical analysis of consecutive liver sections from mice fed a high-fat high-cholesterol diet (HFHC) for 16 weeks. Serial sections were independently stained for Chi3l1 and lineage markers: HNF4α (hepatocytes), Desmin (hepatic stellate cells [HSCs]), or Iba1 (hepatic macrophages). Cellular localization was determined by aligning morphological profiles across sequential sections. Scale bars: 20 μm (main panels), 5 μm (insets). Hepatocytes are outlined in Chi3l1^+^ images in green dashed lines. (**B**) Chi3l1 expression in KCs was assessed by co-staining with F4/80 (red) and TIM4 (white) in livers of mice fed either normal chow diet (NCD, 0 weeks) or HFHC (16 weeks). Scale bar: 20 μm (main panels), 5 μm (inset). The intensity of Chi3l1 expression in KCs was quantified. Unpaired Student’s t-test (**B**). p-Value as indicated. Figure 6—figure supplement 1—source data 1.Numerical data of Figure 6—figure supplement 1B.

**Figure 6—figure supplement 2.**
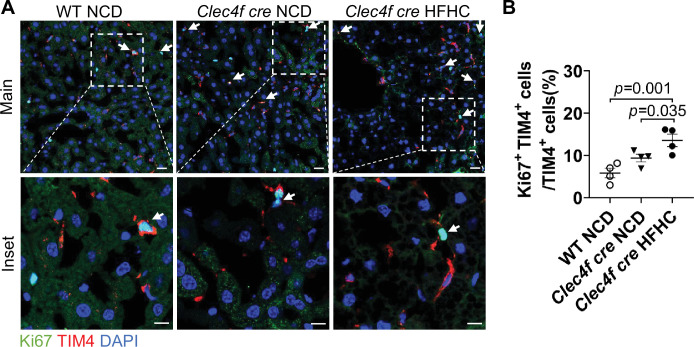
Cre insertion promotes Kupffer cell (KC) self-proliferation. (**A**) Comparision of KC self-proliferation in wild-type (WT) or Clec4f cre mice under normal chow diet (NCD) or Clec4f cre mice under high-fat high-cholesterol diet (HFHC) feeding by co-staining of Ki67 (proliferation marker) and TIM4 (KC marker). Nuclei are counterstained with DAPI (blue). Scale bar: 20 μm (main panels) and 10 μm (inset). (**B**) KC proliferation was quantified. Representative images are shown in A. One-way ANOVA (**B**). p-Value as indicated. Figure 6—figure supplement 2—source data 1.Numerical data of Figure 6—figure supplement 2B.

To determine cell-autonomous effects, we generated KC-specific *Chi3l1* knockout mice (Chi3l1-KpKO mice) and analyzed their response to HFHC feeding. Flow cytometry of NPCs revealed a significantly lower KC population (CD45^+^ F4/80^hi^ CD11b^low^ TIM4^+^) in Chi3l1-KpKO mice compared to *Clec4f cre* controls after 16 weeks of HFHC (Figure 6D and E). During this analysis, we observed no reduction in KCs in *Clec4f cre* control mice, raising the possibility that Cre insertion itself might influence KC maintenance. To test this, we performed co-staining for TIM4 and Ki67, which revealed significantly higher numbers of proliferating KCs in *Clec4f cre* mice compared with C57BL/6J mice under NCD. Notably, following HFHC feeding, *Clec4f cre* mice exhibited an even greater increase in KC proliferation (Figure 6—figure supplement 2A and B), suggesting that cre insertion enhanced KC self-renewal, which contributes to maintenance of the KC pool in these mice under MASLD. Despite this proliferative baseline, Chi3l1-KpKO mice still displayed a reduced KC number (Figure 6D and E) and increased KC death. This was corroborated by TIM4/TUNEL co-staining, which showed elevated KC death in Chi3l1-KpKO livers (Figure 6F and G). Collectively, these findings demonstrate that genetic ablation of *Chi3l1* amplifies glycolytic flux and sensitizes KCs to lipotoxic stress in vitro, and drives KC depletion through accelerated cell death in vivo. Although Chi3l1 is not exclusively expressed by KCs, these data establish it as a critical regulator of KC survival in MASLD, likely acting in a cell-autonomous manner within the KC population.

## Discussion

**Figure 7.**
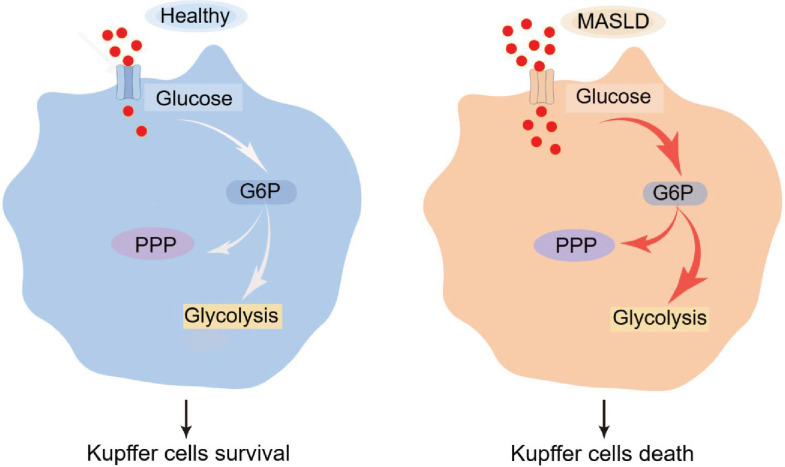
Excessive glycolysis enhancement promotes Kupffer cell (KC) death in metabolic dysfunction-associated steatotic liver disease (MASLD). (Left) Under physiological conditions, KCs maintain basal glucose metabolism supporting cellular homeostasis and survival. (Right) During MASLD progression, KCs undergo excessive glycolysis enhancement, which accelerates KC death.

This study identifies KC death as an early and selective pathological feature of MASLD across multiple dietary models. Compared with other hepatic cell populations, KCs exhibit markedly increased susceptibility to apoptosis during early disease. Through metabolomic and functional analyses, we demonstrate that KCs undergo progressive metabolic reprogramming characterized by sustained activation of glycolysis. Importantly, our data support a causal role for excessive glycolytic flux in driving KC death. Pharmacologic glycolytic stimulation (high glucose or PDK1 activation with PS48), enforced glycolytic dependence (oligomycin), and KC-specific Chi3l1 deletion each accelerated KC apoptosis, whereas glycolytic inhibition (2-DG) was protective. Together, these findings establish hyperactivation of glycolysis as a central mechanism underlying KC vulnerability and depletion in MASLD (Figure 7).

KCs loss has been reported to varying degrees across MASLD models, reflecting differences in diet composition, disease duration, macrophage heterogeneity, and marker selection. For example, in a 6-week MCD model, approximately 10% of CLEC4F^+^ KCs were TUNEL^+^, indicating apoptotic death (Tran et al., 2020). In contrast, another 6-week MCD study reported a marked reduction in TIM4^+^ KCs, from 66% to 26% (Zhang et al., 2024). More moderate KC loss has been observed in HFD models, with an ~20% reduction of TIM4^+^ KCs after 16 weeks (Daemen et al., 2021), whereas prolonged Western diet feeding resulted in a >50% decrease in TIM4^+^ KCs at 36 weeks (Daemen et al., 2021). Together, these studies underscore the model-dependent nature of KC loss and highlight the importance of experimental context and marker selection when assessing KC dynamics in MASLD. Importantly, our spatial analyses further reveal preferential periportal KC death during early MASLD. Given the metabolic zonation of the liver and higher periportal glucose flux, this regional susceptibility supports a mechanistic link between glucose metabolism and KC survival. These findings suggest that metabolic microenvironment, in addition to inflammatory signaling, shapes KC fate during MASLD progression.

While glycolytic reprogramming is classically associated with macrophage activation (Tacke, 2017), our data reveal a critical distinction in tissue-resident KCs. In MASLD, sustained and excessive glycolytic activation does not simply maintain inflammatory function but instead drives apoptotic loss. Thus, glycolysis in this context becomes maladaptive, serving as a determinant of cell fate rather than merely an activation program. This shifts the conceptual framework from inflammation-centered KC dysfunction to metabolism-driven KC attrition. Our findings also clarify the context-dependent role of Chi3l1 in liver disease. Prior studies in advanced MASH and fibrotic models have described pro-fibrotic functions of Chi3l1, including activation of HSCs and modulation of macrophage survival pathways (Kim et al., 2023, Higashiyama et al., 2019, Nishimura et al., 2021). In contrast, using an HFHC model that recapitulates steatohepatitis without fibrosis, we identify a distinct early-stage role for Chi3l1 in maintaining KC metabolic homeostasis. KC-specific deletion of Chi3l1, but not broader myeloid deletion, accelerated KC loss and worsened steatosis (He et al., 2025). These results establish Chi3l1 as a KC-autonomous regulator that restrains glycolytic overload. The apparent duality of Chi3l1 function likely reflects disease stage and cellular source: protective in early MASLD through preservation of resident KCs, but pro-fibrotic in advanced disease when derived from MoMFs and acting on stellate cells.

Our prior work demonstrated that KCs exhibit relative metabolic inflexibility compared with MoMFs, relying heavily on glucose utilization (He et al., 2025). The current study extends this concept by showing that loss of Chi3l1 leads to excessive glycolytic flux that exceeds KC metabolic tolerance as supported by ¹³C-glucose tracing, enhanced lipotoxic sensitivity in vitro, and accelerated cell death in vivo. Thus, Chi3l1 functions as a metabolic checkpoint that fine-tunes glucose uptake and prevents lethal glycolytic overload. This selective dependence explains why Chi3l1 deficiency disproportionately affects KCs while sparing other macrophage populations.

Several limitations merit consideration. First, although multiple murine dietary models were used, validation in human MASLD—particularly spatial assessment of KC death and metabolic signatures—is necessary. Second, while we identify glycolytic hyperactivation as an upstream driver, the downstream apoptotic mechanisms remain incompletely defined. Potential contributors include oxidative stress, redox imbalance, LA accumulation, or mitochondrial insufficiency under high glycolytic flux. Third, the interplay between glucose metabolism and other metabolic pathways, including fatty acid oxidation, warrants further investigation.

Despite these limitations, our findings have translational implications. Therapeutic strategies that preserve KC metabolic balance—rather than broadly suppressing inflammation—may represent a novel approach for MASLD. Modulation of Chi3l1 signaling or selective targeting of key glycolytic regulators within KCs could help maintain the resident macrophage pool, preserve hepatic immune homeostasis, and potentially delay disease progression. In summary, we identify excessive glycolytic reprogramming as a fundamental mechanism driving selective KC apoptosis in early MASLD, with periportal predominance. Chi3l1 functions as an endogenous metabolic safeguard that restrains this lethal shift. Targeting KC-specific metabolic vulnerability may offer a new strategy to preserve hepatic immune integrity and modify MASLD progression.

## Materials and methods

### Animal experiments and procedures

#### Animals

*Chi3l1^-/-^* (strain no. T014402), *Chi3l1^flox//flox^* (strain no. T013652), and *Clec4f-cre* (C-type lectin domain family 4-Cre (*Clec4f-cre*), strain no. T036801) mice with a C57BL/6J background were purchased from GemPharmatech. Accordingly, C57BL/6J mice (strain no. N000013) were used as WT mice. To generate Chi3l1-KpKO mice, *Chi3l1^flox//flox^* mice were crossed with *Clec4f-cre* mice, and knockout efficiency was examined in KCs previously (He et al., 2025). All mouse colonies were maintained at the Animal Core Facility of Yunnan University. The animal studies were approved by the Yunnan University Institutional Animal Care and Use Committee (IACUC, Approval No. YNU20220314). Male mice aged 6–8 weeks were used in this study. For 2-DG treatment in mice, WT C57BL/6J mice were fed a HFHC for 5 weeks. Beginning at week 3, mice received intraperitoneal injections of either vehicle or 2-DG (50 mg/kg) every other day for 2 weeks. Mice were then euthanized for analysis of KC viability and liver pathology.

#### Construction of MASLD/MASH mouse model

Mice were provided an HFHC (Research Diet, d12108c, 40 kcal% fat and 1.25% cholesterol) or an HFD (Research Diet, d12492, 60 kcal% fat). Another group of mice was fed an MCD (Research Diet, A02082002BR). Throughout the feeding period, the body weight and food consumption of the mice were observed and recorded weekly. Once the dietary intervention was completed, the mice were euthanized. Liver and murine serum samples were collected for further analysis. ALT and AST levels in the serum, as well as cholesterol (TC) and TG levels in both serum and liver tissues, were quantified using commercially available kits (Nanjing Jiancheng Bioengineering Institute). Genotyping sample preparation and procedure were conducted as previously described (He et al., 2025).

### Isolation of NPCs and KCs

Hepatic NPCs were isolated as previously described (Chen et al., 2024). In brief, the abdominal cavity of mice was promptly opened, and the hepatic portal vein was located after anesthesia. A soft needle was inserted for perfusion with perfusion buffer (100 mL 1× HBSS, 200 µL 0.5 mol/L EGTA) for 3–5 min, while the inferior vena cava was cut to clear the liver. Subsequently, digestion buffer (60 mL 1× HBSS, 120 µL 2 M MgSO_4_, 60 µL 1.25 M CaCl_2_•H_2_O, 0.015 g collagenase I) was injected at a consistent rate for approximately 15 min. Following perfusion, the liver was delicately transferred into a pre-cooled Petri dish with PBS, cut into 2–3 mm fragments, and incubated at 37°C for 15 min in digestion buffer. The liver tissue fluid was then filtered through a 70 µm cell filter and centrifuged at 50×*g* for 5 min (ac/brake = 0) at 4°C to collect the supernatant. This supernatant underwent centrifugation at 450×*g* for 5 min (ac/brake = 5) at 4°C to obtain the NPC population. The NPCs were suspended in a 15 mL centrifuge tube with 4 mL 20% OptiPrep solution, followed by centrifugation at 3000 rpm for 17 min (ac/brake = 0) at 4°C. After centrifugation, the cells at the boundary between the OptiPrep solution and 1× HBSS buffer were collected into a new tube, supplemented with 1× HBSS buffer, and centrifuged for 5 min at 450×*g* (ac/brake = 5). Red blood cells were lysed with 1 mL ACK buffer (150 mM NH4Cl, 10 mM KHCO_3_, 0.1 mM Na_2_EDTA, pH = 7.2–7.4), neutralized with 1× HBSS buffer, and centrifuged. The cells were resuspended in cell medium and placed in a 3.5 cm Petri dish for 10 min to obtain KCs by washing out un-adherent cells with PBS. The purity and viability of isolated KCs reached 90% and 80%, respectively, which was confirmed by staining with anti-TIM4 antibodies or trypan blue. LDH activity in culture supernatants was measured using a commercial assay kit (Promega, Cat# G1780) according to the manufacturer’s instructions. Absorbance was then measured at 490 nm using a microplate reader (Thermo Fisher Scientific, Multiskan Sky).

### KC processing and metabolomic profiling

The extraction method of cellular metabolites was assessed as previously described (Liu et al., 2022). Primary KCs were isolated from livers of HFHC-fed mice at 0, 4, and 8 weeks using established protocols. After a 15 min adherence period, cells were washed twice with ice-cold PBS and detached using 1 mL ice-cold PBS followed by gentle scraping. Cell suspensions were transferred to 1.5 mL tubes, and 3×10⁶ cells were pelleted by centrifugation (1000×*g*, 5 min). Supernatants were discarded, and pellets snap-frozen in liquid nitrogen. Metabolites were extracted from frozen pellets with 80% methanol (vol/vol) and analyzed via LC-MS/MS using an AB Sciex 6500 Plus QTRAP mass spectrometer coupled to an ExionLC system. All metabolomic processing and data analysis were performed by BioDeep (https://www.biodeep.cn). Related data are provided in Supplementary file 1.

### Flow cytometry analysis of NPCs

The NPCs were resuspended in fluorescence-activated cell sorting (FACS) buffer, consisting of PBS supplemented with 2% bovine serum albumin. 1 µL of anti-CD16/CD32 antibody (Invitrogen, Cat# 14-0161-86) was added to the cell suspension, and the mixture was incubated at 4°C for 10 min to block any nonspecific binding. After blocking, the mouse NPCs were labeled with monoclonal antibodies conjugated with fluorescent dyes. This labeling process was carried out at 4°C for 30 min. The labeled cells were washed thrice with cold FACS buffer. The antibodies used for labeling were as follows: anti-mouse F4/80 (APC) (Invitrogen, Cat# 17-4801-82), anti-CD45 (eFluor450) (Invitrogen, Cat# 48-0451-82), anti-mouse TIM4 (PE) (Invitrogen, Cat# 12-5866-82), and anti-mouse CD11b (PerCP/Cyanine5.5) (BioLegend, Cat# 101228). All the primary antibodies were used at a dilution of 1:100. The samples were analyzed by flow cytometry using an LSR Fortessa Cell Analyzer (BD Biosciences). The data obtained were further analyzed using FlowJo version 10.0.

### Preparation of BMDMs

To obtain BMDMs, femur and tibia bone marrow from healthy male C57BL6/J was extracted, resuspended in DMEM/F12 medium (VivaCell, Cat# C3113-0500) containing 10% FBS (VivaCell, Cat# C04001-500) and 20% L929 conditioned medium, seeded in culture dishes, and cultured at 37°C with 5% CO_2_ in a humidified atmosphere for 7 days. Fresh medium was added on day 4. The cells were maintained in a standard 37°C in 5% CO_2_ incubator. L929 was a gift from Dr. Guangxun Meng (Hainan Academy of Medical Sciences). Cell identity has been authenticated by the STR profiling. Mycoplasma contamination was tested negative by PCR.

### Isotope tracing

^13^C-tracing experiments were assessed as previously described (Tong et al., 2024). For ^13^C-tracing experiments, BMDMs were isolated from WT and *Chi3l1^-/-^* mice, cultured to maturity, and replated. After 12 hr, cells were incubated for 12 hr in glucose-depleted medium supplemented with 10% dialyzed FBS and 15 mmol/L universally labeled [U-^13^C]glucose (Cambridge Isotope Laboratories, CLM-1396-1). Cells were subsequently washed twice with ice-cold glucose-free medium. Following supernatant removal, metabolites were extracted using pre-cooled 80% (vol/vol) methanol for cell lysis.

Metabolite separation was performed using a Vanquish UHPLC system (Thermo Fisher Scientific) equipped with an Amide column (Waters). The mobile phase consisted of: (A) 10 mM ammonium acetate and 0.3% ammonia in water, and (B) 10 mM ammonium acetate and 0.3% ammonia in 90% acetonitrile. Metabolites were separated using a linear gradient elution. Metabolites were ionized, and mass spectrometry data were acquired using an Orbitrap Fusion Tribrid mass spectrometer (Thermo Fisher Scientific). Targeted metabolite analysis was performed by LC-MS/MS using a QTRAP 5500+ system (SCIEX) coupled to an ExionLC AD UHPLC system (SCIEX). Isotope tracing analysis and metabolomic data processing were conducted by BioDeep using the BioDeep Platform (https://www.biodeep.cn). Related data are provided in Supplementary file 2.

### Histology and immunofluorescence H&E staining

Tissues were fixed with buffered 10% paraformaldehyde (Sangon Biotech, Cat# A500684-0500) overnight at 4°C and embedded in paraffin. Ultrathin tissue slices (5 μm) were prepared and deparaffinized. H&E staining was performed on the tissue sections, and the slides were examined under a microscope (Olympus, BP80).

### Immunofluorescence on frozen section

Immunofluorescence staining was conducted on frozen sections of fresh liver tissues. Initially, the tissues were fixed using 2% paraformaldehyde for 1 hr and subsequently dehydrated overnight in a 30% sucrose solution. The following day, tissue embedding was performed using OCT (SAKURA, Cat# 4583), after which ultrathin sections of 5 μm were sliced. Permeabilization was achieved using 0.02% Triton X-100 for 10 min at room temperature, followed by blocking with 5% normal goat serum (VivaCell, Cat# C2530-0100).

#### Co-staining of cleaved caspase 3 with Clec4f

The sections were incubated with primary antibodies, including anti-mouse Clec4f (BioLegend, Cat# 156804, 1:300, Alexa Fluor 647) and Cleaved caspase 3 (Cell Signaling, Cat# 9664S, 1:300). The secondary antibodies used were 488-conjugated Affinipure Goat Anti-Rabbit IgG (H+L) (Jackson ImmunoResearch, 111-545-003, 1:600) to label the Cleaved caspase 3. Nuclei were stained with DAPI (Beyotime, c1006, prediluted), and images were captured using a confocal laser scanning microscope (ZEISS, LSM900).

#### Co-staining of TIM4 and F4/80 with Chi3l1

The sections were incubated with primary antibodies, including anti-mouse TIM4 (BioLegend, Cat# 130008, 1:300, Alexa Fluor 647), anti-mouse F4/80 (BioLegend, Cat# 123140, 1:300, Alexa Fluor 594), and Chi3l1 (Abcam, ab180569, 1:400). The secondary antibodies used were 488-conjugated Affinipure Goat Anti-Rabbit IgG (H+L) (Jackson ImmunoResearch, 111-545-003, 1:600) to label the Chi3l1. Nuclei were stained with DAPI (Beyotime, c1006, prediluted), and images were captured using a confocal laser scanning microscope (ZEISS, LSM900).

#### Co-staining of Clec4f, TIM4, HNF4a, Desmin, IBA1, GS, and Ki67 with TUNEL

TUNEL staining was performed on separate frozen sections of liver tissues. Following fixation with 4% paraformaldehyde and treatment with Proteinase K (20 μg/mL in PBS, BBI, Cat# B600169-0002), the sections were permeabilized with Triton X-100 and blocked with normal goat serum. Subsequently, the sections were incubated with an anti-mouse Clec4f antibody (BioLegend, Cat# 156804, 1:300, Alexa Fluor 647), anti-mouse TIM4 (BioLegend, Cat# 130008, 1:300, Alexa Fluor 647), HNF4α (Abcam, Cat# ab181604, 1:400), Desmin (Proteintech, Cat# 15620-1-AP, 1:400), IBA1 (Fujifilm, Cat# 019-19741, 1:300), GS (Proteintech, Cat# 11037-2-AP, 1:400), and Ki67 (Abcam, Cat# ab15580, 1:300). The secondary antibodies used were 488-conjugated Affinipure Goat Anti-Rabbit IgG (H+L) (Jackson ImmunoResearch, 111-545-003, 1:600), followed by TUNEL staining (Servicebio, Cat# G1502-100T) according to the manufacturer’s instructions. Nuclei were counterstained with DAPI, and images were captured using a confocal laser scanning microscope (ZEISS, LSM900).

### Immunohistochemical localization of Chi3l1

Liver frozen sections from male C57BL/6J mice fed an HFHC for 16 weeks were sectioned serially (3 µm). Consecutive sections were independently stained using standard immunofluorescence protocols for: Chi3l1 (Abcam, ab180569, 1:400). Lineage markers: HNF4α (Abcam, Cat# ab181604, 1:400), Desmin (Proteintech, Cat# 15620-1-AP, 1:400), Iba1 (Fujifilm, Cat# 019-19741, 1:300). The secondary antibodies used were 488-conjugated Affinipure Goat Anti-Rabbit IgG (H+L) (Jackson ImmunoResearch, 111-545-003, 1:600). Nuclei were counterstained with DAPI, and images were captured using a confocal laser scanning microscope (ZEISS, LSM900). Cellular localization was assigned by aligning morphological features across sequentially stained sections using nuclear and cytoplasmic landmarks.

### Immunofluorescent staining on KCs

KCs were seeded in 24-well plates and subjected to various treatments. In one set of experiments, cells were treated with either DMSO (1 μL, Sangon Biotech, Cat# A100231-0500) as a control or oligomycin (1 mM in 1 μL, Abcam, Cat# ab141829) for 24 hr. Another experiment involved treatment with DMSO or PS48 (20 mM in 1 μL, Sigma, Cat# P0022) for the same duration. Additionally, BMDMs were treated with different glucose concentrations: 5.5 mM glucose alone, 5.5 mM glucose with PA (800 mM in 1 μL, Sigma, Cat# P0500), or 10 mM glucose with PA, all for 24 hr. The cell slides were washed with cold PBS and fixed with 4% paraformaldehyde in PBS for 10 min at room temperature. The cells were permeabilized with 0.02% Triton X-100 for 10 min at room temperature. After blocking with 5% normal goat serum, cells were incubated with primary antibodies anti-Cleaved caspase 3 (Cell Signaling, Cat# 9664S, 1:300) overnight at 4°C. On the second day, after washing with 0.05% PBST, cells were incubated with 488-conjugated Goat Anti-Rabbit IgG (H+L) (Jackson ImmunoResearch, 111-545-003, 1:1000) for 1 hr at room temperature. After rinsing with 0.05% PBST, cells were counterstained with DAPI (Beyotime, C1006) and mounted onto slides. Images were captured using a confocal laser scanning microscope (ZEISS, LSM900). The identification of KCs follows the above-mentioned experimental protocol. Primary antibodies against TIM4 (BioLegend, Cat# 130008, 1:300, Alexa Fluor 647) are employed to label KCs.

#### Calcein-AM staining on KCs

Live-cell staining using Calcein-AM was conducted following the manufacturer’s instructions from a commercially available kit (Beyotime, Cat# C2015M). KCs were seeded in 12-well plates and subjected to various treatments. In one set of experiments, cells were treated with either DMSO (1 μL, Sangon Biotech, Cat# A100231-0500) as a control or oligomycin (1 mM in 1 μL, Abcam, Cat# ab141829) for 24 hr. Another experiment involved treatment with DMSO or PS48 (20 mM in 1 μL, Sigma, Cat# P0022) for the same duration. Additionally, BMDMs were treated with different glucose concentrations: 5.5 mM glucose alone, 5.5 mM glucose with PA (800 mM in 1 μL, Sigma, Cat# P0500), or 10 mM glucose with PA, all for 24 hr.

#### Oil Red O staining

Oil Red O staining was conducted on unfixed frozen sections embedded directly in OCT. Frozen sections of the liver were cut at a thickness of 10 μm. After rinsing with water, the sections were immersed in 60% isopropanol for 2 min. Subsequently, the sections were stained with an Oil Red O staining solution (Solarbio, Cat# IO1720) at 37°C for 10–15 min. Following staining, the sections were immediately placed in 60% isopropanol and washed three to five times to eliminate excess dye solution. Nuclei were counterstained with a hematoxylin staining solution. After rinsing with distilled water, the sections were sealed with glycerol gelatin (Solarbio, Cat# S2150).

#### Sirius Red staining

Tissues were fixed with buffered 10% paraformaldehyde (Sangon Biotech, Cat# A500684-0500) overnight at 4°C and embedded in paraffin. Ultrathin tissue slices (5 μm) were prepared and deparaffinized. Sirius Red staining (Solarbio, Cat# G1472) was performed according to the manufacturer’s instructions, and the slides were examined under a microscope (Olympus, BP80).

### Spatial analysis of KC death

To evaluate the spatial distribution of KC death along the portal-central axis, the distance between the portal vein (PV) and central vein (CV) was measured in histological sections. The PV-CV axis was systematically divided into three equidistant zones (periportal, intermediate, and pericentral) for regional analysis. KC death was quantified by isolating fluorescence signals corresponding to cell death markers (TUNEL) through channel thresholding and noise reduction. The positive area and integrated fluorescence intensity were measured within each zone, excluding vascular structures to focus on parenchymal KC populations. This zonal approach enabled comparative assessment of KC death patterns across different hepatic microenvironments.

### Diagnosis of MASLD activity score

Murine MASLD activity was assessed histologically using the NAFLD Activity Score (NAS) on H&E-stained liver sections based on the following features: hepatocyte ballooning degeneration (0–2), lobular inflammation (0–3), and steatosis grade (0–3) (Brunt et al., 2011). The individual scores were summed to yield the total NAS (range 0–8) per animal. A NAS≥5 was considered diagnostic for steatohepatitis (MASH), NAS≤3 indicated not-MASH, and NAS=4 was indeterminate.

### ECAR measurement

The ECAR measurements were conducted according to the manufacturer’s instructions (Agilent, Cat# 103020-100). Briefly, BMDMs were cultured in a Seahorse XF24 cell culture plate. After 12 hr of culture, the DMEM culture medium was pre-treated for 24 hr. 1 hr before the analysis, the culture medium was changed to the corresponding XF basal medium (Agilent, Cat# 103334-100) supplemented with glutamine (the final concentration was 2 mmol/L, Agilent, Cat# 103579-100), and the culture plates were incubated at 37°C without CO_2_. Compounds were added in the following order: 10 mM glucose, 1.0 mM oligomycin, and 50 mM 2-deoxyglucose. Measurements were conducted using a Seahorse XF24 analyzer (Agilent Technologies). After completing the Seahorse XF Glycolytic Stress Test, basic glycolytic ability and total glycolytic ability were calculated based on the generated report.

### LDH release assay for cytotoxicity measurement

The LDH measurements were conducted according to the manufacturer’s instructions (Promega, Cat# G183A). An appropriate amount of cells were inoculated into the culture plate and treated according to the experimental plan. At the scheduled detection time, 50 μL of supernatant from each well was added to a 96-well plate, and then 50 μL of working solution was added to each well, and incubated at room temperature in the dark for 30 min. Immediately after the reaction, 50 μL of termination solution was added to each well to terminate the reaction. Immediately thereafter, the absorbance of the reaction was measured at 490 nm using an enzyme-labeled instrument (Thermo Scientific).

### Western blot analysis

The sample preparation and procedure were conducted as previously described (Chen et al., 2024). The following antibodies were used: anti-Caspase 3 (Cell Signaling Technology, Cat# 9662S, 1:1000), anti-Cleaved caspase 3 (Cell Signaling Technology, Cat# 9664S, 1:1000), anti-β-actin (Proteintech, Cat# 66009-1-lg, 1:1000). Peroxidase-conjugated Affinipure Goat Anti-Mouse IgG (H+L) (Jackson ImmunoResearch, 115-035-003, 1:2000) and Peroxidase-conjugated Affinipure Goat Anti-Rabbit IgG (H+L) (Jackson ImmunoResearch, 111-035-003, 1:2000) were used for secondary antibody incubation.

### RNA extraction and quantitative real-time PCR

The TRIzol reagent (Invitrogen, Cat# 15596018) was used to lyse cells or ground tissues, and the resulting mixture was centrifuged at 12,000 rpm/min at 4°C for 10 min. The liquid portion (supernatant) was carefully transferred to a new tube. To this tube, 200 μL of chloroform was added and mixed thoroughly. The tube was left at room temperature for 10 min and subsequently centrifuged. The resulting supernatant was collected in a new tube. Isopropanol, in the same volume as the supernatant, was added to the tube and mixed thoroughly. After incubating for 10 min at 4°C, the mixture was centrifuged. The supernatant was then carefully discarded. RNA was precipitated by adding 75% ethanol to the tube, followed by centrifugation. This process was repeated. The tube was dried for 10 min at room temperature, and an appropriate amount of ribonuclease-free water was added to dissolve the RNA precipitate. The concentration and purity of RNA samples were determined using an ultraviolet spectrophotometer (NanoDrop). The RNA template was subjected to reverse transcription using a cDNA first-strand synthesis kit (Takara, Cat# 6210B). Quantitative PCR was performed using SYBR Green Master Mix (Thermo Fisher, Cat# A25742) in triplicates following the manufacturer’s instructions. This was performed on a Real-Time PCR QuatStudio1 with accompanying software, following the instructions provided by the manufacturer (Life Technologies, Grand Island, NY, USA). Primer sequences are provided in Supplementary file 3.

### Data presentation and statistical analysis

Data in graph figures are presented as mean ± standard error of the mean (SEM). Statistical analyses were performed using SPSS Statistics (version 22). For comparisons between two groups, an unpaired two-tailed Student’s t-test was used, while one-way analysis of variance (ANOVA) was applied for comparisons involving three or more groups. A p-value<0.05 was considered statistically significant, with p-values indicated where applicable. All cell culture experiments were repeated at least three times independently. Figure 7 was created using the Figdraw platform (https://www.figdraw.com).

## Data Availability

All data generated or analysed during this study are included in the manuscript and supporting files; source data files have been provided.

## Additional files

Supplementary file 1. Metabolomics data of primary Kupffer cells from high-fat high-cholesterol diet (HFHC)-fed mice at 0, 4, and 8 weeks.

Supplementary file 2. Isotope tracing and metabolomics data for [U-¹³C]glucose.

Supplementary file 3. qPCR primers.

MDAR checklist

## Appendix 1

**Appendix 1—key resources table:**

Reagent type (species) or resource	Designation	Source or reference	Identifiers	Additional information
Antibody	Rat monoclonal anti-F4/80 (APC) antibody	Invitrogen	Cat# 17-4801-82; RRID:AB_2784648	Flow cytometry (1:100)
Antibody	Rat monoclonal anti-CD45 (eFluor450) antibody	Invitrogen	Cat# 48-0451-82; RRID:AB_1518806	Flow cytometry (1:100)
Antibody	Rat monoclonal anti-TIM-4 (PE) antibody	Invitrogen	Cat# 12-5866-82; RRID:AB_1257163	Flow cytometry (1:100)
Antibody	Rat monoclonal Anti-CD16/CD32	Invitrogen	Cat# 14-0161-86; RRID:AB_467135	Flow cytometry (1:100)
Antibody	Rat monoclonal anti-CD11b (PerCP/Cyanine5.5) antibody	BioLegend	Cat# 101228; RRID:AB_893232	Flow cytometry (1:100)
Antibody	Ly-6G monoclonal antibody (1A8-Ly6g) PE-Cyanine7	Invitrogen	Cat# 25-9668-82; RRID:AB_2811793	Flow cytometry (1:100)
Antibody	Mouse monoclonal anti-Clec4f (Alexa Fluor 647) antibody	BioLegend	Cat# 156804; RRID:AB_2814082	IF (1:300)
Antibody	Mouse monoclonal anti-TIM4 (Alexa Fluor 647) antibody	BioLegend	Cat# 130008; RRID:AB_2271648	IF (1:300)
Antibody	Mouse monoclonal anti-β-actin antibody	Proteintech	Cat# 66009–1-lg; RRID:AB_2687938	WB (1:1000)
Antibody	Cleaved caspase 3 rabbit antibody	Cell Signaling Technology	Cat# 9664S; RRID:AB_2070042	IF (1:300), WB (1:1000)
Antibody	Caspase 3 rabbit antibody	Cell Signaling Technology	Cat# 9662S; RRID:AB_331439	WB (1:1000)
Antibody	HNF4α rabbit antibody	Abcam	Cat# ab181604; RRID:AB_2890918	IF (1:400)
Antibody	Desmin Polyclonal antibody	Proteintech	Cat#16520-1-AP	IF (1:400)
Antibody	IBA1 rabbit antibody	Fujifilm	Cat# 019-19741; RRID:AB_839504	IF (1:300)
Antibody	Glutamine Synthetase Polyclonal antibody	Proteintech	Cat#11037-2-AP	IF (1:400)
Antibody	Ki67 Rabbit antibody	Abcam	Cat# ab15580; RRID:AB_443209	IF (1:300)
Antibody	Rabbit polyclonal Anti-YKL-40/CHI3L1 antibody	Abcam	Cat# ab180569; RRID:AB_2891040	IF (1:400)
Antibody	Peroxidase-conjugated Affinipure Goat Anti-Rabbit IgG (H+L)	Jackson	Cat# 111-035-003; RRID:AB_2313567	WB (1:2000)
Antibody	Peroxidase-conjugated Affinipure Goat Anti-Mouse IgG (H+L)	Jackson	Cat# 115-035-003; RRID:AB_10015289	WB (1:2000)
Antibody	Alexa Fluor 488-conjugated Affinipure Goat Anti-Mouse IgG+IgM (H+L)	Jackson	Cat# 115-545-044; RRID:AB_2338844	IF (1:600)
Antibody	Alexa Fluor 568-goat anti-rabbit IgG (H+L) cross-adsorbed secondary antibody	Invitrogen	Cat# A11011; RRID:AB_143157	IF (1:600)
Chemical compound, drug	FBS	VivaCell	Cat# C04001-500	
Chemical compound, drug	Phosphate Buffered Saline	VivaCell	Cat# C3580-0500	
Chemical compound, drug	DMEM (High glucose)	VivaCell	Cat# C3113-0500	
Chemical compound, drug	DMEM (No glucose)	Sigma	Cat# D5030	
Chemical compound, drug	^13^C-Glucose	Cambridge Isotope Laboratories	Cat# CLM-1396-1	
Chemical compound, drug	Dialysis fetal bovine serum	Abcam	Cat# DIA0500	
Chemical compound, drug	Sodium pyruvate	Sangon Biotech	Cat# A501259-0100	
Chemical compound, drug	Penicillin-Streptomycin Solution	VivaCell	Cat# C3421-0100	
Chemical compound, drug	Cell Dissociation Solution	Sartorius	Cat# 03-079-1B	
Chemical compound, drug	β-Mercaptoethanol	Sigma	Cat# M3148	
Chemical compound, drug	Eosin Y (water soluble)	Aladdin	Cat# E141405	
Chemical compound, drug	Hematoxylin	BBI	Cat# A600701-0050	
Chemical compound, drug	Oil Red O	Solarbio	Cat# IO1720	
Chemical compound, drug	Sirius Red	Sangon Biotech	Cat# A500684-0500	
Chemical compound, drug	High-effect paraffin-ceresin	Shanghai Hualing Rehabilitation Equipment Manufacturing Plant	Cat# N/A	
Chemical compound, drug	10% Neutral Formalin Fix Solution	BBI	Cat# E672001-0500	
Chemical compound, drug	Xylene	Tianjin Zhiyuan Chemical Reagents Co., Ltd.	Cat# N/A	
Chemical compound, drug	Neutral balsam	Solarbio	Cat# G8590	
Chemical compound, drug	Isopropanol	Sangon Biotech	Cat# A507048-0500	
Chemical compound, drug	Tissue-Tek OCT compound	SAKURA	Cat# REF:4583	
Chemical compound, drug	Paraformaldehyde	Sangon Biotech	Cat# A500684-0500	
Chemical compound, drug	Acetone	Chron Chemicals	Cat# N/A	
Chemical compound, drug	Sucrose	Sangon Biotech	Cat# A502792-0005	
Chemical compound, drug	Triton X-100	BBI	Cat# A600198-0500	
Chemical compound, drug	Goat serum	VivaCell	Cat# C2530-0100	
Chemical compound, drug	Tween 20	BBI	Cat# A600560-0500	
Chemical compound, drug	DAPI Staining Solution	Beyotime	Cat# C1006	
Chemical compound, drug	Antifade Mounting Medium with DAPI	Beyotime	Cat# P0131	
Chemical compound, drug	Omni-Easy One-Step PAGE Gel Fast Preparation Kit	Epizyme	Cat# PG213	
Chemical compound, drug	SDS	BBI	Cat# A600485-0500	
Chemical compound, drug	Glycine	BBI	Cat# A502065-0005	
Chemical compound, drug	Tris	Solarbio	Cat# T8060	
Chemical compound, drug	Methanol	Ghtech	Cat# N/A	
Chemical compound, drug	NON-Fat Powdered Milk	BBI	Cat# NON-Fat Powdered Milk	
Chemical compound, drug	Collagenase, Type 1	Diamond	Cat# A004194-0001	
Chemical compound, drug	Cytiva Percoll Centrifugation Media	Cytiva	Cat# 17089101	
Chemical compound, drug	Heparin sodium from Porcine Intestinal	Sangon Biotech	Cat# A603251-0001	
Chemical compound, drug	1 M HEPES	Solarbio	Cat# H1095	
Chemical compound, drug	OptiPrep	Serumwerk Bernburg	Cat# 1893	
Chemical compound, drug	DNase I, RNase-free	Thermo	Cat# EN0521	
Chemical compound, drug	CaCl_2_	GHTECH	Cat#10043-52-4	
Chemical compound, drug	MgSO_4_·7H_2_O	Sangon Biotech	Cat# A610329-0500	
Chemical compound, drug	TRIzol reagent	Invitrogen	Cat# 15596018	
Chemical compound, drug	UltraPure DNase/RNase-Free Distilled Water	Invitrogen	Cat# 10977015	
Chemical compound, drug	Trichloromethane	Chron Chemicals	Cat# N/A	
Chemical compound, drug	PowerUp SYBR Green Master Mix	Applied Biosystems	Cat# A25742	
Chemical compound, drug	DEPC水	Biosharp	Cat# 701062	
Chemical compound, drug	MgCl_2_	GHTECH	Cat# N/A	
Chemical compound, drug	KCl	Sangon Biotech	Cat# A501159-0500	
Chemical compound, drug	NaHCO_3_	Sangon Biotech	Cat# A500873-0500	
Chemical compound, drug	NaOH	BBI	Cat# A620617-0500	
Chemical compound, drug	2-DG	Sigma	Cat# D8375	
Chemical compound, drug	PS48	Sigma	Cat# P0022	
Chemical compound, drug	Palmitic acid	Sigma	Cat# P0500	
Chemical compound, drug	DMSO	Sangon Biotech	Cat# A100231-0500	
Chemical compound, drug	Proteinase K Solution (20 mg/mL)	BBI	Cat# B600169-0002	
Chemical compound, drug	Glycerol Gelatin aqueous slide mounting medium	Solarbio	Cat# S2150	
Chemical compound, drug	XF basal medium	Agilent	Cat#103334-100	
Chemical compound, drug	XF 200 mmol/L Glutamine solution	Agilent	Cat#103579-100	
Chemical compound, drug	BD Pharmingen Stain Bu­er (FBS)	BD Biosciences	Cat# 554656	
Chemical compound, drug	Draq7	BD Biosciences	Cat# 564904	
Chemical compound, drug	High-fat rodent diet with 1.25% cholesterol	Research Diet	Cat# d12108c	
Chemical compound, drug	Methionine and choline-deficient diet	Research Diet	Cat# A02082002BR	
Chemical compound, drug	Proteinase K Solution (20 mg/mL)	BBI	Cat# B600169-0002	
Chemical compound, drug	Glycerol Gelatin aqueous slide mounting medium	Solarbio	Cat# S2150	
Chemical compound, drug	XF basal medium	Agilent	Cat#103334-100	
Chemical compound, drug	XF 200 mmol/L Glutamine solution	Agilent	Cat#103579-100	
Chemical compound, drug	BD Pharmingen Stain Bu­er (FBS)	BD Biosciences	Cat# 554656	
Chemical compound, drug	Draq7	BD Biosciences	Cat# 564904	
Chemical compound, drug	High-fat rodent diet with 1.25%cholesterol	Research Diet	Cat# d12108c	
Chemical compound, drug	Methionine and choline-deficient diet	Research Diet	Cat# A02082002BR	
Peptide, recombinant protein	Recombinant mouse Chi3l1	SB	Cat# 50929M08H	
Commercial assay or kit	TMR (red) TUNEL Cell Apoptosis Detection Kit	Servicebio	Cat# G1502-100T	
Commercial assay or kit	Calcein/PI Cell Viability /Cytotoxicity Assay Kit	Beyotime	Cat# C2015M	
Commercial assay or kit	Alanine aminotransferase assay kit	Nanjing Jiancheng Bioengineering Institute	Cat# C009-2-1	
Commercial assay or kit	Aspartate aminotransferase assay kit	Nanjing Jiancheng Bioengineering Institute	Cat# C010-2-1	
Commercial assay or kit	Total cholesterol assay kit	Nanjing Jiancheng Bioengineering Institute	Cat# A111-1-1	
Commercial assay or kit	Triglyceride assay kit	Nanjing Jiancheng Bioengineering Institute	Cat# A110-1-1	
Commercial assay or kit	Seahorse XF Glycolysis Stress Test Kit	Agilent	Cat# 103020-100	
Commercial assay or kit	PrimeScript II 1st Strand cDNA Synthesis Kit	TaKaRa	Cat# 6210B	
Commercial assay or kit	CytoTox 96Non-Radioactive Cytotoxicity Assay	Promega	Cat# G183A	
Software, algorithm	GraphPad Prism	GraphPad software	RRID:SCR_002798	https://www.graphpad.com
Software, algorithm	FlowJo V10	FlowJo Software	RRID:SCR_008520	https://www.flowjo.com/
Software, algorithm	ImageJ	National Institutes of Health	RRID:SCR_003070	https://imagej.nih.gov/ij/
Software, algorithm	SPSS	IBM SPSS software	RRID:SCR_002865	https://www.ibm.com/
Software, algorithm	Seahorse Wave	Agilent Technologies	RRID:SCR_024491	https://www.agilent.com/
Software, algorithm	ZEN microscope software	ZEISS	RRID:SCR_013672	https://www.zeiss.com.cn/
Cell line (*Mus musculus*, male)	NCTC clone 929 (L-929)	ATCC	CCL-1	L929 was a gift from Dr. Guangxun Meng (Hainan Academy of Medical Sciences)
Strain, strain background (*Mus musculus*, male)	Chi3l1^-/-^	GemPharmatech Co., Ltd.	T014402	Genetic modification: constitutive knockout
Strain, strain background (*Mus musculus*, male)	Chi3l1^flox/flox^	GemPharmatech Co., Ltd.	T013652	Genetic modification: floxed allele (homozygous)
Strain, strain background (*Mus musculus*, male)	Clec4f cre	GemPharmatech Co., Ltd.	T036801	Genetic modification: Cre recombinase transgene under Clec4f promoter
Other	Bone marrow-derived macrophage (BMDM)	This paper	N/A	Strain: C57BL/6J
Other	Kupffer cell (KC)	This paper	N/A	Strain: C57BL/6J

## References

[bib1] Ben-Moshe S, Veg T, Manco R, Dan S, Papinutti D, Lifshitz A, Kolodziejczyk AA, Bahar Halpern K, Elinav E, Itzkovitz S (2022). The spatiotemporal program of zonal liver regeneration following acute injury. Cell Stem Cell.

[bib2] Brunt EM, Kleiner DE, Wilson LA, Belt P, Neuschwander-Tetri BA, NASH Clinical Research Network (CRN) (2011). Nonalcoholic fatty liver disease (NAFLD) activity score and the histopathologic diagnosis in NAFLD: distinct clinicopathologic meanings. Hepatology.

[bib3] Chen Y, Yang R, Qi B, Shan Z (2024). Peptidoglycan-Chi3l1 interaction shapes gut microbiota in intestinal mucus layer. eLife.

[bib4] Daemen S, Gainullina A, Kalugotla G, He L, Chan MM, Beals JW, Liss KH, Klein S, Feldstein AE, Finck BN, Artyomov MN, Schilling JD (2021). Dynamic shifts in the composition of resident and recruited macrophages influence tissue remodeling in NASH. Cell Reports.

[bib5] Du L, Lin L, Li Q, Liu K, Huang Y, Wang X, Cao K, Chen X, Cao W, Li F, Shao C, Wang Y, Shi Y (2019). IGF-2 preprograms maturing macrophages to acquire oxidative phosphorylation-dependent anti-inflammatory properties. Cell Metabolism.

[bib6] Eslam M, Newsome PN, Sarin SK, Anstee QM, Targher G, Romero-Gomez M, Zelber-Sagi S, Wai-Sun Wong V, Dufour J-F, Schattenberg JM, Kawaguchi T, Arrese M, Valenti L, Shiha G, Tiribelli C, Yki-Järvinen H, Fan J-G, Grønbæk H, Yilmaz Y, Cortez-Pinto H, Oliveira CP, Bedossa P, Adams LA, Zheng M-H, Fouad Y, Chan W-K, Mendez-Sanchez N, Ahn SH, Castera L, Bugianesi E, Ratziu V, George J (2020a). A new definition for metabolic dysfunction-associated fatty liver disease: An international expert consensus statement. Journal of Hepatology.

[bib7] Eslam M, Sanyal AJ, George J, Panel IC (2020b). MAFLD: a consensus-driven proposed nomenclature for metabolic associated fatty liver disease. Gastroenterology.

[bib8] Faas M, Ipseiz N, Ackermann J, Culemann S, Grüneboom A, Schröder F, Rothe T, Scholtysek C, Eberhardt M, Böttcher M, Kirchner P, Stoll C, Ekici A, Fuchs M, Kunz M, Weigmann B, Wirtz S, Lang R, Hofmann J, Vera J, Voehringer D, Michelucci A, Mougiakakos D, Uderhardt S, Schett G, Krönke G (2021). IL-33-induced metabolic reprogramming controls the differentiation of alternatively activated macrophages and the resolution of inflammation. Immunity.

[bib9] Friedman SL, Neuschwander-Tetri BA, Rinella M, Sanyal AJ (2018). Mechanisms of NAFLD development and therapeutic strategies. Nature Medicine.

[bib10] Gomez Perdiguero E, Klapproth K, Schulz C, Busch K, Azzoni E, Crozet L, Garner H, Trouillet C, de Bruijn MF, Geissmann F, Rodewald H-R (2015). Tissue-resident macrophages originate from yolk-sac-derived erythro-myeloid progenitors. Nature.

[bib11] Hardy T, Oakley F, Anstee QM, Day CP (2016). Nonalcoholic fatty liver disease: pathogenesis and disease spectrum. Annual Review of Pathology.

[bib12] Hashimoto D, Chow A, Noizat C, Teo P, Beasley MB, Leboeuf M, Becker CD, See P, Price J, Lucas D, Greter M, Mortha A, Boyer SW, Forsberg EC, Tanaka M, van Rooijen N, García-Sastre A, Stanley ER, Ginhoux F, Frenette PS, Merad M (2013). Tissue-resident macrophages self-maintain locally throughout adult life with minimal contribution from circulating monocytes. Immunity.

[bib13] He J, Chen B, Wang X, Yang R, Deng C, Zhu X, Wang K, Wang L, Lu X, Peng C, Li C, Shan Z (2025). Differential regulation of hepatic macrophage fate by Chi3l1 in MASLD. eLife.

[bib14] Higashiyama M, Tomita K, Sugihara N, Nakashima H, Furuhashi H, Nishikawa M, Inaba K, Wada A, Horiuchi K, Hanawa Y, Shibuya N, Kurihara C, Okada Y, Nishii S, Mizoguchi A, Hozumi H, Watanabe C, Komoto S, Yamamoto J, Seki S, Miura S, Hokari R (2019). Chitinase 3-like 1 deficiency ameliorates liver fibrosis by promoting hepatic macrophage apoptosis. Hepatology Research.

[bib15] Kazankov K, Jørgensen SMD, Thomsen KL, Møller HJ, Vilstrup H, George J, Schuppan D, Grønbæk H (2019). The role of macrophages in nonalcoholic fatty liver disease and nonalcoholic steatohepatitis. Nature Reviews. Gastroenterology & Hepatology.

[bib16] Kim AD, Kui L, Kaufmann B, Kim SE, Leszczynska A, Feldstein AE (2023). Myeloid-specific deletion of chitinase-3-like 1 protein ameliorates murine diet-induced steatohepatitis progression. Journal of Molecular Medicine.

[bib17] Liu Q, Zhu F, Liu X, Lu Y, Yao K, Tian N, Tong L, Figge DA, Wang X, Han Y, Li Y, Zhu Y, Hu L, Ji Y, Xu N, Li D, Gu X, Liang R, Gan G, Wu L, Zhang P, Xu T, Hu H, Hu Z, Xu H, Ye D, Yang H, Li B, Tong X (2022). Non-oxidative pentose phosphate pathway controls regulatory T cell function by integrating metabolism and epigenetics. Nature Metabolism.

[bib18] Ma J, Wei K, Liu J, Tang K, Zhang H, Zhu L, Chen J, Li F, Xu P, Chen J, Liu J, Fang H, Tang L, Wang D, Zeng L, Sun W, Xie J, Liu Y, Huang B (2020). Glycogen metabolism regulates macrophage-mediated acute inflammatory responses. Nature Communications.

[bib19] Murray PJ, Allen JE, Biswas SK, Fisher EA, Gilroy DW, Goerdt S, Gordon S, Hamilton JA, Ivashkiv LB, Lawrence T, Locati M, Mantovani A, Martinez FO, Mege J-L, Mosser DM, Natoli G, Saeij JP, Schultze JL, Shirey KA, Sica A, Suttles J, Udalova I, van Ginderachter JA, Vogel SN, Wynn TA (2014). Macrophage activation and polarization: nomenclature and experimental guidelines. Immunity.

[bib20] Nishimura N, De Battista D, McGivern DR, Engle RE, Tice A, Fares-Gusmao R, Kabat J, Pomerenke A, Nguyen H, Sato S, Bock KW, Moore IN, Kleiner DE, Zamboni F, Alter HJ, Govindarajan S, Farci P (2021). Chitinase 3-like 1 is a profibrogenic factor overexpressed in the aging liver and in patients with liver cirrhosis. PNAS.

[bib21] Okada J, Landgraf A, Xiaoli AM, Liu L, Horton M, Schuster VL, Yang F, Sidoli S, Qiu Y, Kurland IJ, Eliscovich C, Shinoda K, Pessin JE (2025). Spatial hepatocyte plasticity of gluconeogenesis during the metabolic transitions between fed, fasted and starvation states. Nature Metabolism.

[bib22] Remmerie A, Martens L, Thoné T, Castoldi A, Seurinck R, Pavie B, Roels J, Vanneste B, De Prijck S, Vanhockerhout M, Binte Abdul Latib M, Devisscher L, Hoorens A, Bonnardel J, Vandamme N, Kremer A, Borghgraef P, Van Vlierberghe H, Lippens S, Pearce E, Saeys Y, Scott CL (2020). Osteopontin expression identifies a subset of recruited macrophages distinct from kupffer cells in the fatty liver. Immunity.

[bib23] Rinella ME, Sanyal AJ (2016). Management of NAFLD: a stage-based approach. Nature Reviews Gastroenterology & Hepatology.

[bib24] Scott CL, Zheng F, De Baetselier P, Martens L, Saeys Y, De Prijck S, Lippens S, Abels C, Schoonooghe S, Raes G, Devoogdt N, Lambrecht BN, Beschin A, Guilliams M (2016). Bone marrow-derived monocytes give rise to self-renewing and fully differentiated Kupffer cells. Nature Communications.

[bib25] Scott CL, T’Jonck W, Martens L, Todorov H, Sichien D, Soen B, Bonnardel J, De Prijck S, Vandamme N, Cannoodt R, Saelens W, Vanneste B, Toussaint W, De Bleser P, Takahashi N, Vandenabeele P, Henri S, Pridans C, Hume DA, Lambrecht BN, De Baetselier P, Milling SWF, Van Ginderachter JA, Malissen B, Berx G, Beschin A, Saeys Y, Guilliams M (2018). The transcription factor ZEB2 is required to maintain the tissue-specific identities of macrophages. Immunity.

[bib26] Sheka AC, Adeyi O, Thompson J, Hameed B, Crawford PA, Ikramuddin S (2020). Nonalcoholic Steatohepatitis: A Review. JAMA.

[bib27] Tacke F (2017). Targeting hepatic macrophages to treat liver diseases. Journal of Hepatology.

[bib28] Tong L, Chen Z, Li Y, Wang X, Yang C, Li Y, Zhu Y, Lu Y, Liu Q, Xu N, Shao S, Wu L, Zhang P, Wu G, Wu X, Chen X, Fang J, Jia R, Xu T, Li B, Zheng L, Liu J, Tong X (2024). Transketolase promotes MAFLD by limiting inosine-induced mitochondrial activity. Cell Metabolism.

[bib29] Tran S, Baba I, Poupel L, Dussaud S, Moreau M, Gélineau A, Marcelin G, Magréau-Davy E, Ouhachi M, Lesnik P, Boissonnas A, Le Goff W, Clausen BE, Yvan-Charvet L, Sennlaub F, Huby T, Gautier EL (2020). Impaired kupffer cell self-renewal alters the liver response to lipid overload during non-alcoholic steatohepatitis. Immunity.

[bib30] Woods PS, Kimmig LM, Sun KA, Meliton AY, Shamaa OR, Tian YF, Cetin-Atalay R, Sharp WW, Hamanaka RB, Mutlu GM (2022). HIF-1α induces glycolytic reprograming in tissue-resident alveolar macrophages to promote cell survival during acute lung injury. eLife.

[bib31] Younossi Z, Anstee QM, Marietti M, Hardy T, Henry L, Eslam M, George J, Bugianesi E (2018). Global burden of NAFLD and NASH: trends, predictions, risk factors and prevention. Nature Reviews Gastroenterology & Hepatology.

[bib32] Younossi ZM (2019). Non-alcoholic fatty liver disease - A global public health perspective. Journal of Hepatology.

[bib33] Zhang C-S, Li M, Wang Y, Li X, Zong Y, Long S, Zhang M, Feng J-W, Wei X, Liu Y-H, Zhang B, Wu J, Zhang C, Lian W, Ma T, Tian X, Qu Q, Yu Y, Xiong J, Liu D-T, Wu Z, Zhu M, Xie C, Wu Y, Xu Z, Yang C, Chen J, Huang G, He Q, Huang X, Zhang L, Sun X, Liu Q, Ghafoor A, Gui F, Zheng K, Wang W, Wang Z-C, Yu Y, Zhao Q, Lin S-Y, Wang Z-X, Piao H-L, Deng X, Lin S-C (2022). The aldolase inhibitor aldometanib mimics glucose starvation to activate lysosomal AMPK. Nature Metabolism.

[bib34] Zhang J, Wang Y, Fan M, Guan Y, Zhang W, Huang F, Zhang Z, Li X, Yuan B, Liu W, Geng M, Li X, Xu J, Jiang C, Zhao W, Ye F, Zhu W, Meng L, Lu S, Holmdahl R (2024). Reactive oxygen species regulation by NCF1 governs ferroptosis susceptibility of Kupffer cells to MASH. Cell Metabolism.

