# Supplementary figures and images for: Hyperactivated glycolysis drives spatially patterned Kupffer cell depletion in MASLD
Source: eLife. 2026 May 26;14:RP109206. doi: 10.7554/eLife.109206 (PMC13211875; doi:10.7554/eLife.109206)

## Raw unedited membranes

**Figure 4B**

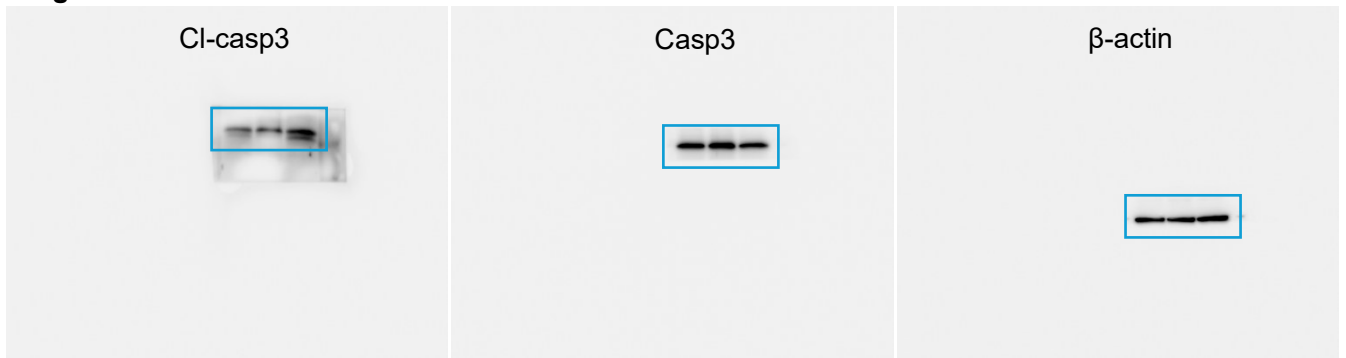

**Figure 4D**

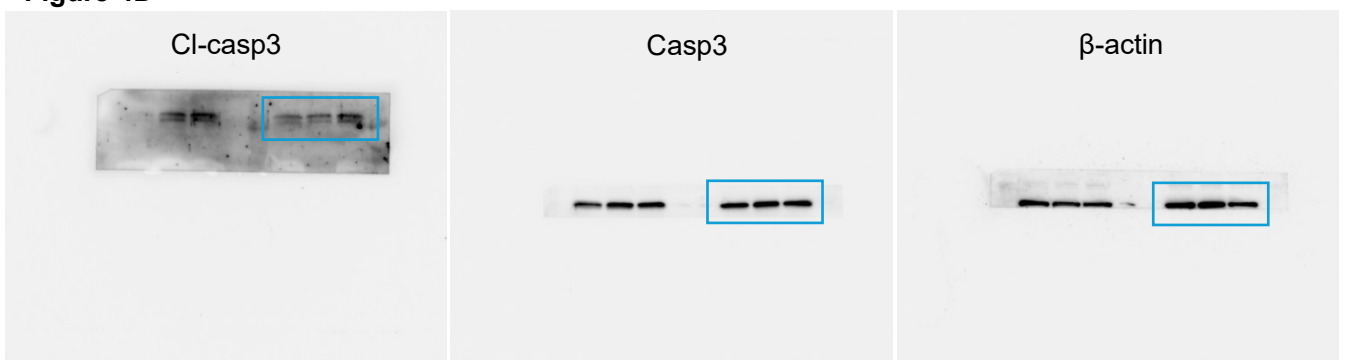

Supplement: Figure 4—source data 2. [file elife-109206-fig4-data2.pdf]

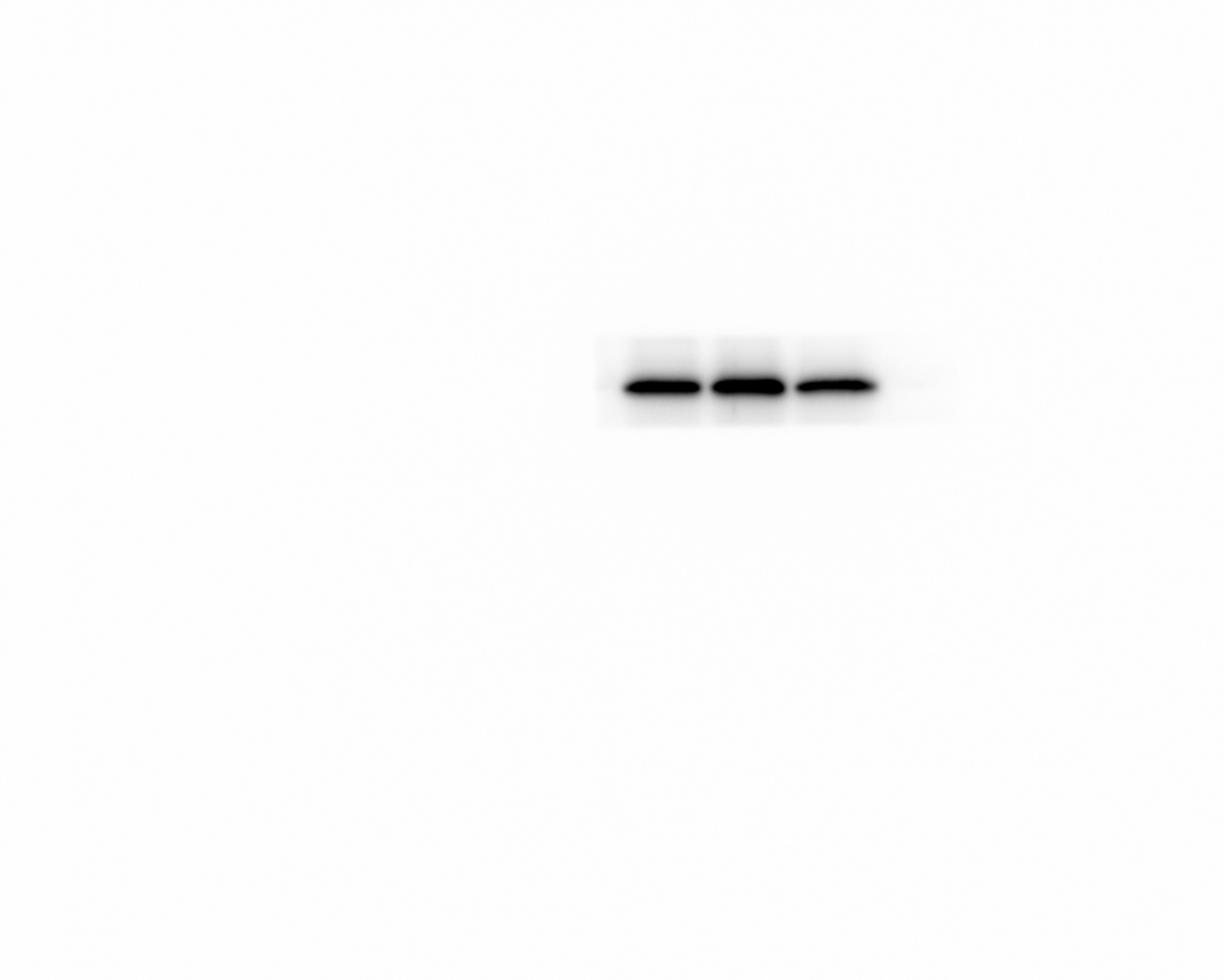

Supplement: Figure 4—source data 3. [file elife-109206-fig4-data3.zip › Figure 4-source data 3/Figure 4B-Casp 3.tif]

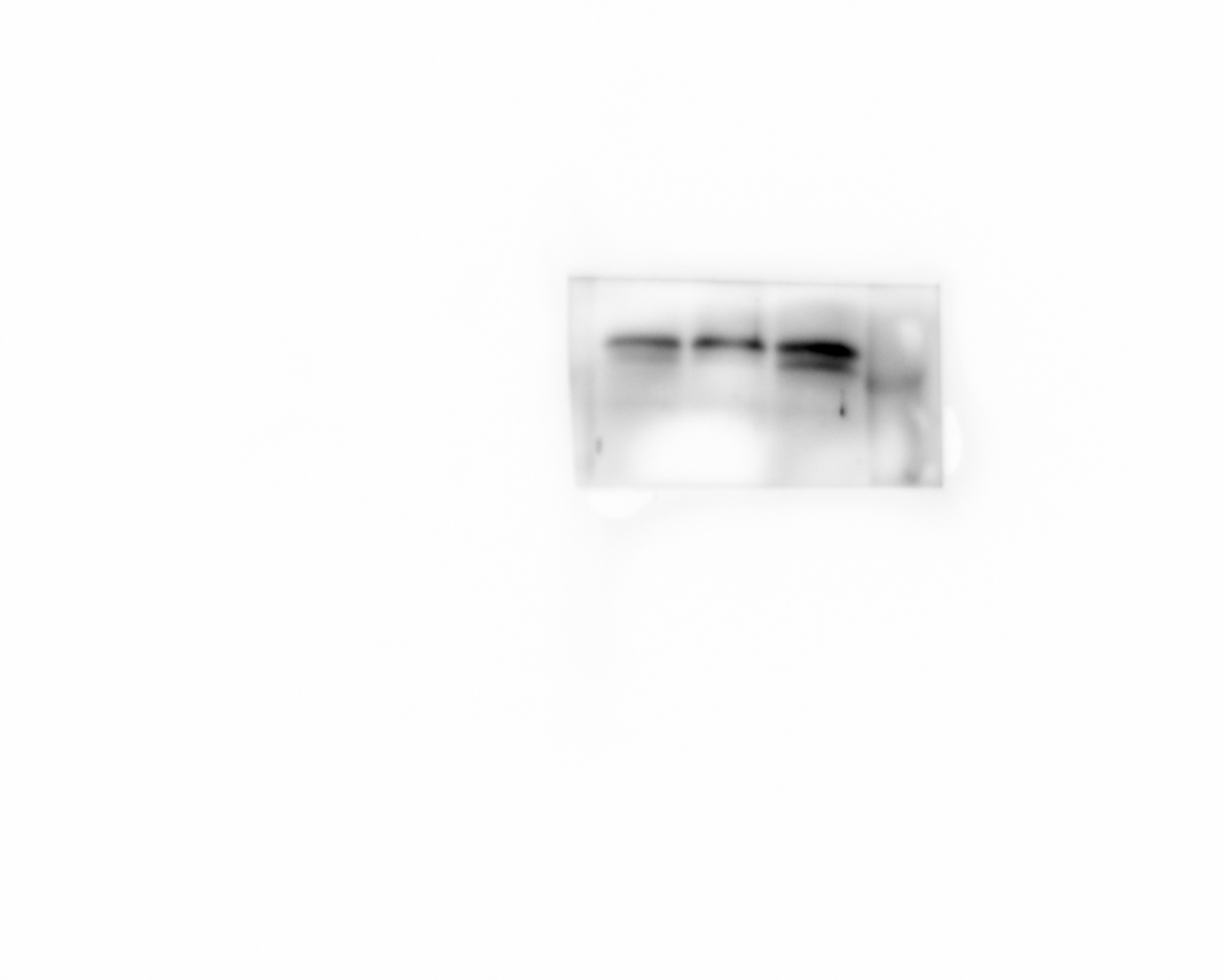

Supplement: Figure 4—source data 3. [file elife-109206-fig4-data3.zip › Figure 4-source data 3/Figure 4B-Cl-casp 3.tif]

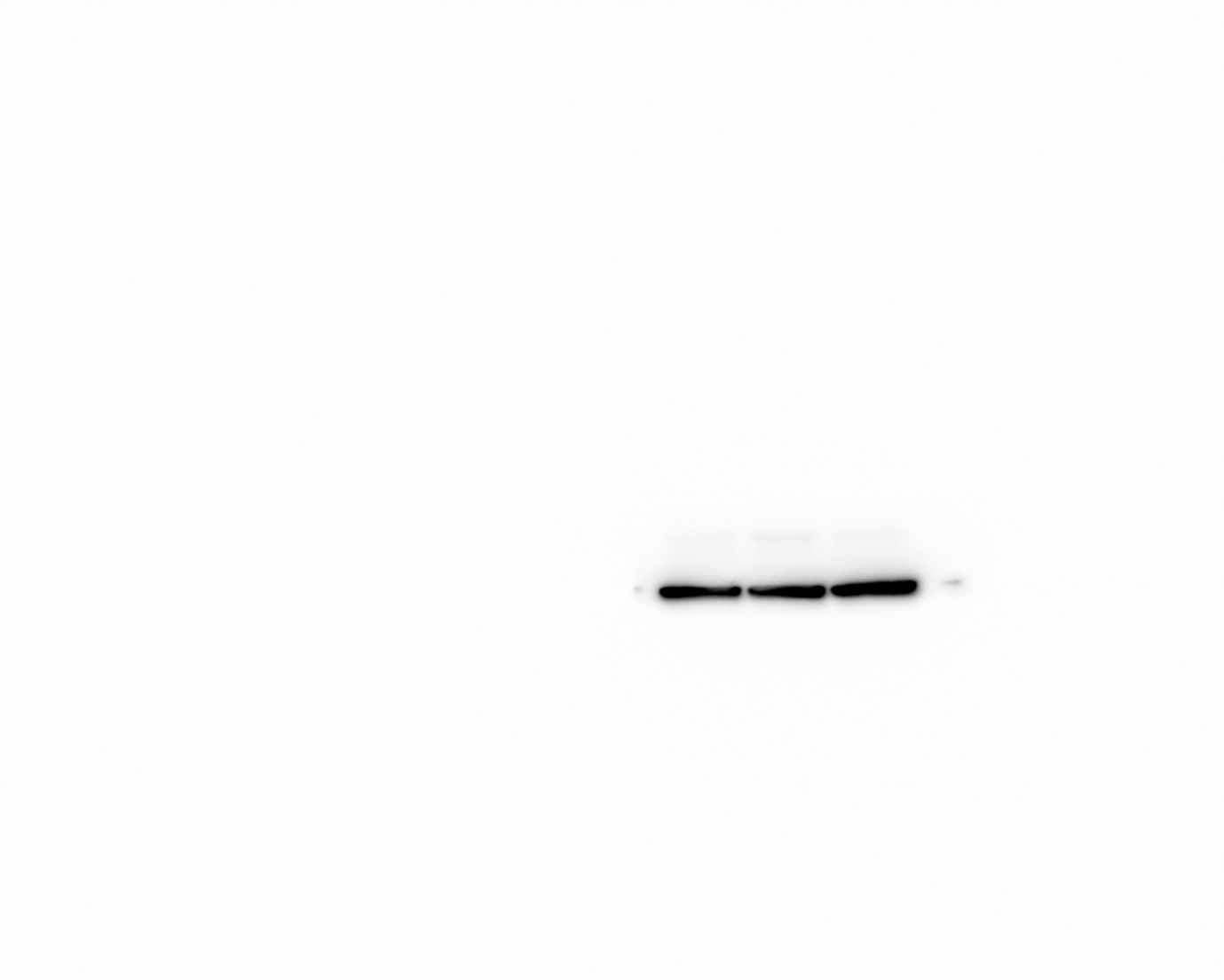

Supplement: Figure 4—source data 3. [file elife-109206-fig4-data3.zip › Figure 4-source data 3/Figure 4B-a┬-actin.tif]

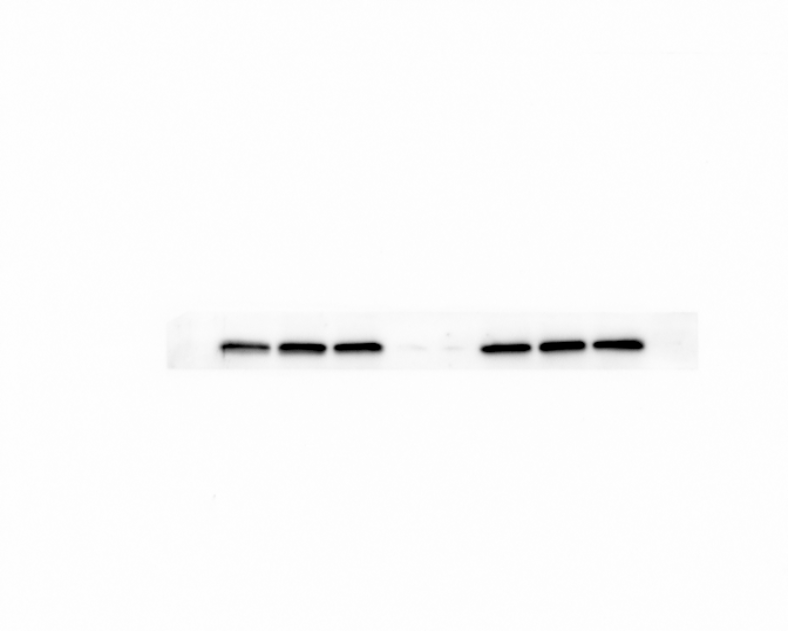

Supplement: Figure 4—source data 3. [file elife-109206-fig4-data3.zip › Figure 4-source data 3/Figure 4D-Casp 3.tif]

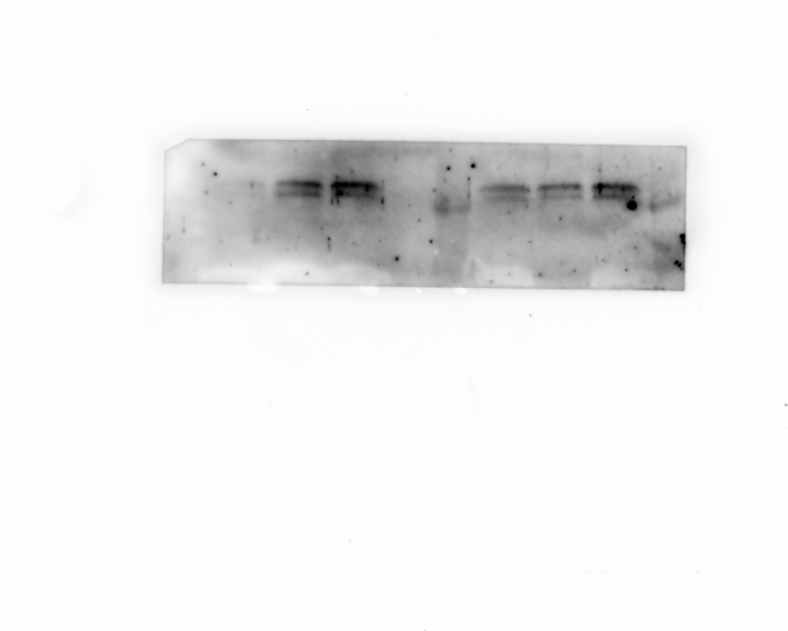

Supplement: Figure 4—source data 3. [file elife-109206-fig4-data3.zip › Figure 4-source data 3/Figure 4D-Cl-casp 3.tif]

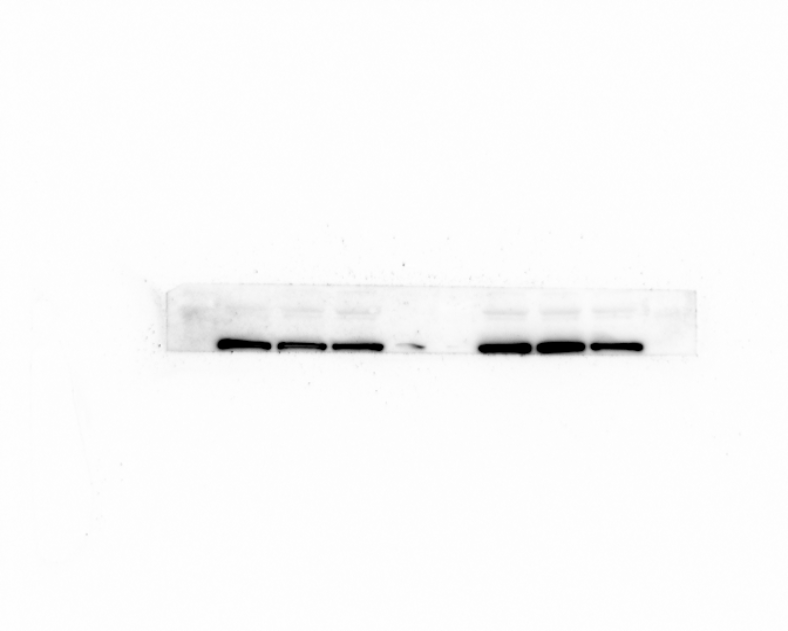

Supplement: Figure 4—source data 3. [file elife-109206-fig4-data3.zip › Figure 4-source data 3/Figure 4D-a┬-actin.tif]

# Raw unedited membranes

Figure 4-Figure supplement 1

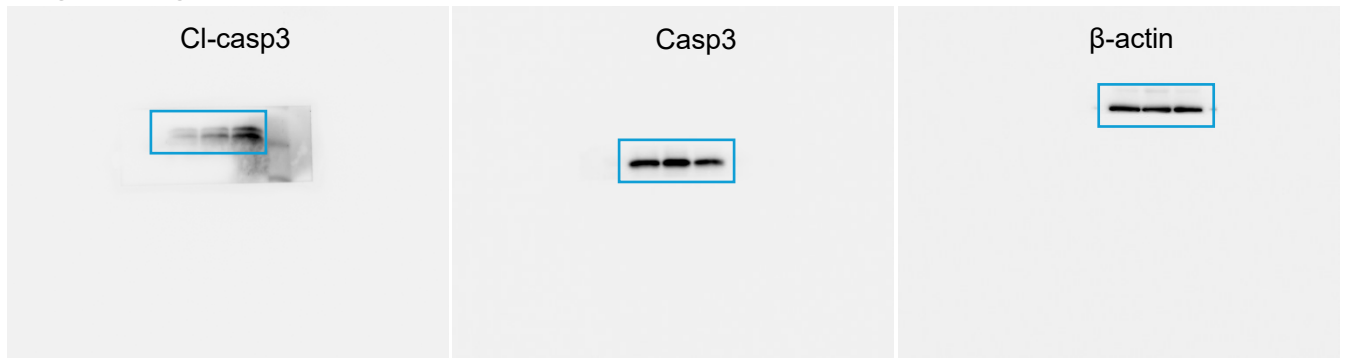

Supplement: Figure 4—figure supplement 1—source data 2. [file elife-109206-fig4-figsupp1-data2.pdf]

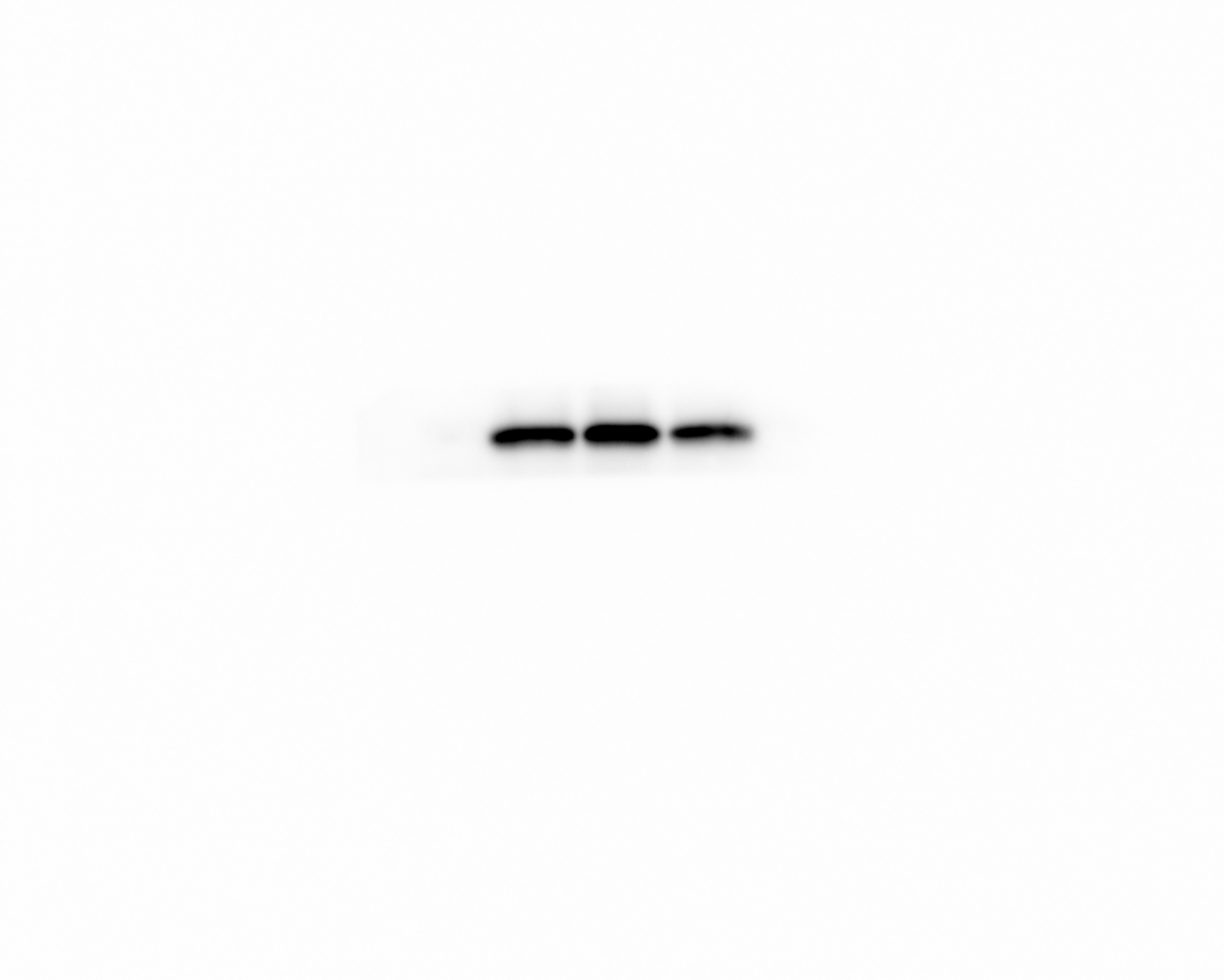

Supplement: Figure 4—figure supplement 1—source data 3. [file elife-109206-fig4-figsupp1-data3.zip › Figure 4-Figure supplement 1-source data 6/Figure 4-Figure supplement 1C-Casp3.tif]

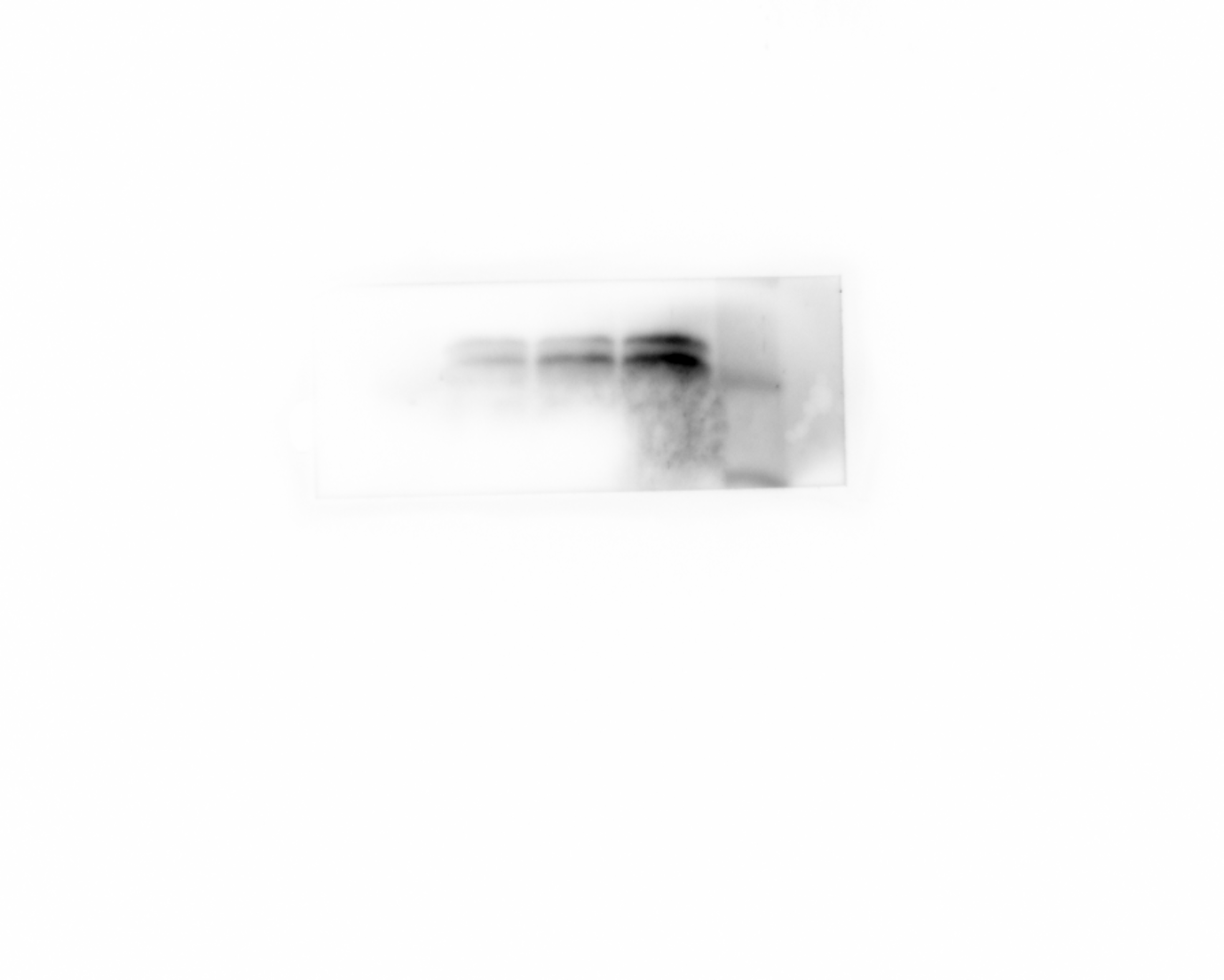

Supplement: Figure 4—figure supplement 1—source data 3. [file elife-109206-fig4-figsupp1-data3.zip › Figure 4-Figure supplement 1-source data 6/Figure 4-Figure supplement 1C-Cl-casp3.tif]

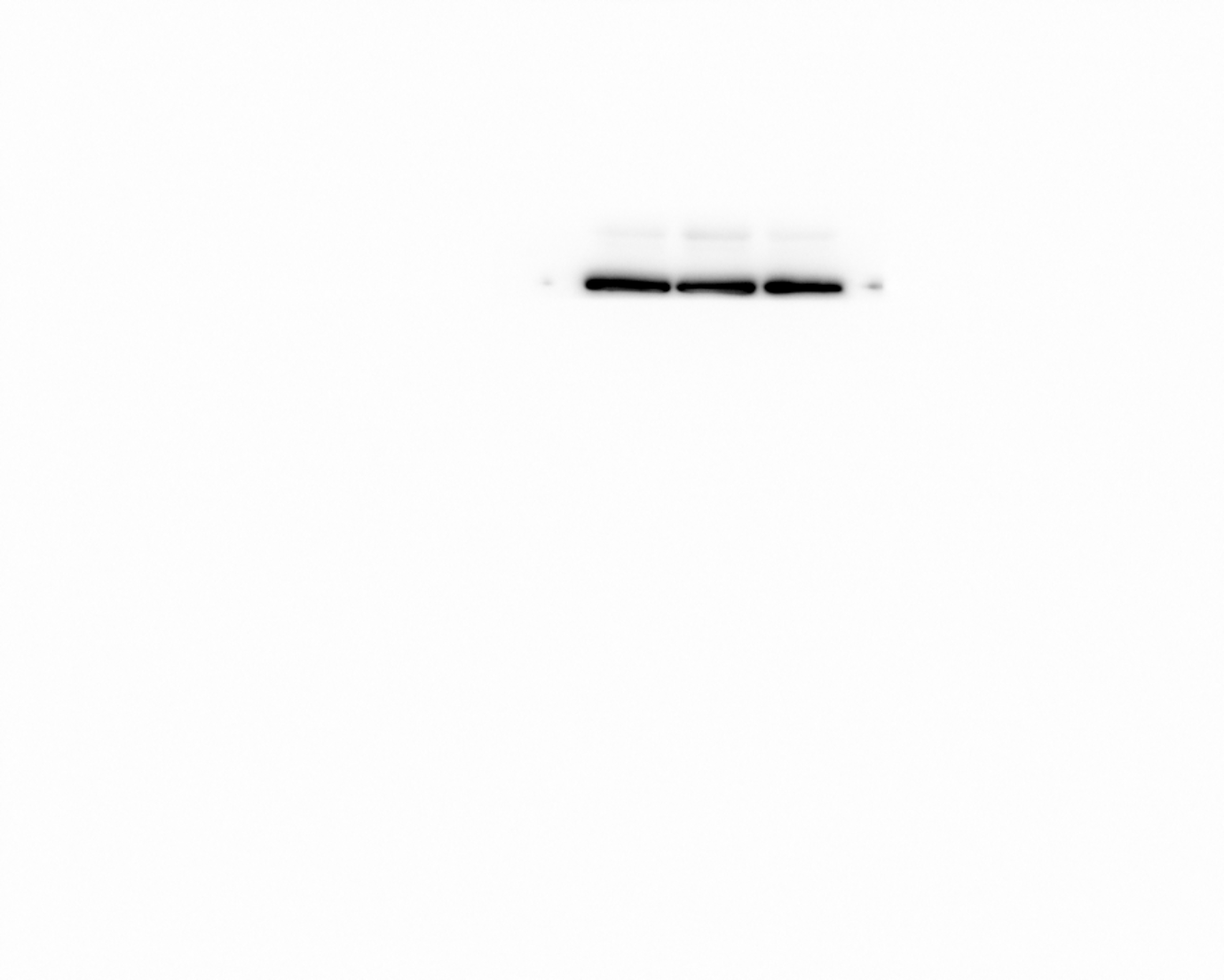

Supplement: Figure 4—figure supplement 1—source data 3. [file elife-109206-fig4-figsupp1-data3.zip › Figure 4-Figure supplement 1-source data 6/Figure 4-Figure supplement 1C-a┬-actin.tif]
