# Supplementary material for: Hyperactivated glycolysis drives spatially patterned Kupffer cell depletion in MASLD
Source: eLife. 2026 May 26;14:RP109206. doi: 10.7554/eLife.109206 (PMC13211875; doi:10.7554/eLife.109206)
Supplement: Supplementary file 3. [file elife-109206-supp3.docx]

**Supplementary file 3**

**qPCR primers**

| Targeted gene | Primer | Sequence |
| --- | --- | --- |
| musSlc2a1 | F(5‘-3’) | CAGTTCGGCTATAACACTGGTG |
|  | R(5‘-3’) | GCCCCCGACAGAGAAGATG |
| musHk3 | F(5‘-3’) | CTGAGTCAAGGCTGTATCCTCC |
|  | R(5‘-3’) | TGCACCAGTTCAGCATCTGAGG |
| musPfkfb3 | F(5‘-3’) | CCCAGAGCCGGGTACAGAA |
|  | R(5‘-3’) | GGGGAGTTGGTCAGCTTCG |
| musPkm | F(5‘-3’) | GCCGCCTGGACATTGACTC |
|  | R(5‘-3’) | CCATGAGAGAAATTCAGCCGAG |
| mus6pgd | F(5‘-3’) | CATCGCTGCAAAAGTGGGAACC |
|  | R(5‘-3’) | AGCCTCACAGATGAGCTGCATG |
| musG6pd | F(5‘-3’) | GACCAAGAAGCCTGGCATGTTC |
|  | R(5‘-3’) | AGACATCCAGGATGAGGCGTTC |
| musPygl | F(5‘-3’) | GGCAGAAGTGGTGAACAATGACC |
|  | R(5‘-3’) | TCCGATAGGTCTGTGGCTGGAA |
| musUgp2 | F(5‘-3’) | CTGATGAACCCACCCAATGGGA |
|  | R(5‘-3’) | GAGCGATTTCCACCAGTCTCAG |
| musGys1 | F(5‘-3’) | CACAGAACGGTTGTCGGACTTG |
|  | R(5‘-3’) | AGGTGAAGTGGTCTGGAAAGGC |
| musIdh1 | F(5‘-3’) | CAGGCTCATAGATGACATGGTGG |
|  | R(5‘-3’) | CACTGGTCATCATGCCAAGGGA |
| musOgdh | F(5‘-3’) | GGTGTCGTCAATCAGCCTGAGT |
|  | R(5‘-3’) | ATCCAGCCAGTGCTTGATGTGC |
| musAcadm | F(5‘-3’) | AGGGTTTAGTTTTGAGTTGACGG |
|  | R(5‘-3’) | CCCCGCTTTTGTCATATTCCG |
| musHadh | F(5‘-3’) | TTCCAGAGGCTGGACAAGTTCG |
|  | R(5‘-3’) | GCCAGCAAATCGGTCTTGTCTG |
| musNos2 | F(5‘-3’) | GAGACAGGGAAGTCTGAAGCAC |
|  | R(5‘-3’) | CCAGCAGTAGTTGCTCCTCTTC |
| musCxcl9 | F(5‘-3’) | CCTAGTGATAAGGAATGCACGATG |
|  | R(5‘-3’) | CTAGGCAGGTTTGATCTCCGTTC |
| musCIITA | F(5‘-3’) | ACCTTCGTCAGACTGGCGTTGA |
|  | R(5‘-3’) | GCCATTGTATCACTCAAGGAGGC |
| MusCD86 | F(5‘-3’) | ACGTATTGGAAGGAGATTACAGCT |
|  | R(5‘-3’) | TCTGTCAGCGTTACTATCCCGC |
| musCcl3 | F(5‘-3’) | ACTGCCTGCTGCTTCTCCTACA |
|  | R(5‘-3’) | ATGACACCTGGCTGGGAGCAAA |
| musCcl5 | F(5‘-3’) | CCTGCTGCTTTGCCTACCTCTC |
|  | R(5‘-3’) | ACACACTTGGCGGTTCCTTCGA |
| musChil3 | F(5‘-3’) | CTCCAGTGTAGCCATCCTTAGG |
|  | R(5‘-3’) | TACTCACTTCCACAGGAGCAGG |
| musRetnla | F(5‘-3’) | CCAAGATCCACAGGCAAAGCCA |
|  | R(5‘-3’) | CAAGGAACTTCTTGCCAATCCAG |
| musArg1 | F(5‘-3’) | GCTGAAGGTCTCTTCCATCACC |
|  | R(5‘-3’) | CATTGGCTTGCGAGACGTAGAC |
| musMrc1 | F(5‘-3’) | GTTCACCTGGAGTGATGGTTCTC |
|  | R(5‘-3’) | AGGACATGCCAGGGTCACCTTT |
